# FedHST: Federated Hybrid Spatial–Temporal Optimization for robust non-IID learning

**DOI:** 10.1371/journal.pone.0360546

**Published:** 2026-11-12

**Authors:** Duy-Dong Le, Thai-Thinh Dang

**Affiliations:** University of Economics Ho Chi Minh City (UEH), Ho Chi Minh City, Vietnam; Jadavpur University, INDIA

## Abstract

Server-side adaptive optimizers in Federated Learning (FL), such as FedAdam and FedAvgM, scale global updates using historical temporal statistics but do not explicitly model instantaneous cross-client variance within a single communication round. Under severe statistical heterogeneity (non-IID data), this can leave the adaptive step size insufficiently damped precisely when local client updates conflict most. We propose **FedHST** (Federated Hybrid Spatial–Temporal Optimization), a server-side aggregation framework that augments the temporal second-moment estimate with an instantaneous, coordinate-wise cross-client spatial variance term. This term acts as an additional damper under high client disagreement and vanishes under consensus, at which point FedHST reduces to standard temporal-only adaptation. We establish a non-convex convergence rate of $𝒪(1/\sqrt{T})$, with the residual term showing explicitly how the spatial damping coefficient γ moderates the effect of statistical heterogeneity on convergence. We evaluate FedHST on FashionMNIST, CIFAR-10, and CIFAR-100 under Dirichlet-partitioned non-IID splits (α∈{0.1,0.3}), comparing against FedAdam and FedDyn — the strongest representatives of the adaptive server-optimization and dynamic client-regularization paradigms, respectively — over three independent random seeds with paired significance testing; FedAvgM, FedYogi, FedAdagrad, FedProx, and SCAFFOLD are reviewed in Related Work. FedHST attains accuracy statistically indistinguishable from, or modestly and significantly exceeding (up to + 0.5 percentage points, *p* < 0.05 on FashionMNIST), FedAdam, while substantially and significantly outperforming FedDyn (+ 5 to + 28 percentage points across datasets, *p* < 0.01), at a computational overhead within 1–4% of FedAdam. Ablation studies further show that FedHST’s advantage over FedAdam grows systematically with the number of local training epochs and with non-IID severity, consistent with the mechanism by which spatial damping is designed to activate under cross-client conflict.

## Introduction

### Federated learning and the non-IID challenge

The growth of data generated at the network edge — by IoT devices, smartphones, and distributed institutional databases — has made it increasingly attractive to train deep learning models directly where the data resides, rather than collecting it centrally. Centralizing raw edge data is often undesirable due to bandwidth constraints and, more importantly, privacy regulations such as the GDPR, CCPA, and HIPAA that restrict the aggregation of sensitive user records. Federated Learning (FL) [1] addresses this by moving computation to the data: a central server coordinates training across many clients, each of which optimizes a shared model on its own private data and returns only model updates, never raw data, to the server [2].

A central obstacle to FL in practice is statistical heterogeneity across clients, commonly termed the non-IID data problem [3,4]: client data distributions can differ in label distribution, feature distribution, or conditional distribution, and in the volume of local data [5]. Under severe heterogeneity — often modeled with a symmetric Dirichlet distribution at a small concentration parameter α [6] — local loss landscapes diverge substantially across clients. When clients perform multiple local epochs (*E* > 1) to reduce communication cost, their models drift toward distinct local optima, a phenomenon termed **client drift** [7]. Naively averaging these drifted models at the server can degrade convergence and increase round-to-round variance in global model performance.

### Spatial blindness in server-side adaptive optimizers

One line of work addresses client drift by modifying server-side aggregation with adaptive, per-coordinate learning rates, most notably the FedOpt family (FedAdam, FedYogi, FedAdagrad) [6], which treats the aggregated client update as a pseudo-gradient and applies coordinate-wise adaptive scaling based on a temporal second-moment estimate $v_{t}$ accumulated across rounds. This formulation, however, characterizes variance only along the *time* axis: at any given round, $v_{t}$ reflects the history of the *mean* aggregated update, and contains no explicit signal about how much individual client updates disagreed with one another *within* that round.

This omission matters most under a specific but plausible failure mode: when opposing client factions produce large updates of similar magnitude but opposite sign for a given coordinate, their weighted mean ${\bar{\Delta }}_{t}$ can approach zero even though the underlying disagreement is severe. Because $v_{t}$ tracks only this mean, it can shrink even as cross-client conflict grows, which — since $v_{t}$ sits in the denominator of the adaptive step — can enlarge the effective step size exactly when client consensus is weakest. We refer to this gap as *spatial blindness*, and it motivates the method proposed in this paper.

### Core research contributions

We present **FedHST** (Federated Hybrid Spatial–Temporal Optimization), a server-side aggregation framework that augments the standard temporal second-moment estimate with an instantaneous, coordinate-wise cross-client spatial variance term, so that the adaptive denominator reflects both historical volatility and present-round disagreement. Our contributions are:

1. **The FedHST optimization engine**: a hybrid spatial-temporal denominator that combines the temporal second moment of global updates with the instantaneous cross-client spatial variance, computable entirely at the server from the pseudo-gradients clients already transmit — requiring no additional client computation, memory, or communication relative to FedAdam.
2. **Non-convex convergence analysis**: we establish a convergence rate of $𝒪(1/\sqrt{T})$, with the residual term explicitly showing how the spatial damping coefficient γ moderates the effect of statistical heterogeneity $\sigma_{H}^{2}$ on the optimization trajectory.
3. **Multi-dataset empirical evaluation with statistical testing**: we evaluate FedHST against FedAdam and FedDyn — selected as the strongest representative of the closely-clustered FedOpt adaptive family and the strongest non-adaptive alternative, respectively (see Results and empirical analysis for the supporting evidence) — on FashionMNIST, CIFAR-10, and CIFAR-100 under Dirichlet non-IID partitioning, across multiple independent seeds with paired significance testing, together with a communication- and computation-overhead analysis. FedAvgM, FedYogi, FedAdagrad, FedProx, and SCAFFOLD are reviewed and positioned relative to FedHST in *Related Work* below.
4. **Ablation studies**: we characterize FedHST’s sensitivity to its spatial damper weight γ, the momentum decay parameters $\beta_{1},\beta_{2}$, client count, participation ratio, local epoch count, and non-IID severity, and show that its advantage over temporal-only adaptive optimizers grows with local epoch count and with non-IID severity — consistent with the mechanism by which the spatial term is designed to activate.

The remainder of this article is organized as follows. Section Related work reviews related federated optimization methods. Section Mathematical preliminaries and problem formulation formalizes the problem. Section The proposed FedHST framework presents FedHST. Section Rigorous Convergence Analysis gives the convergence analysis. Section Experimental setup details the experimental setup. Section Results and empirical analysis reports empirical results and ablation studies. Section Conclusion concludes.

## Related work

The quest to achieve robust numerical convergence and suppress client drift within statistically heterogeneous FL environments has driven intensive research. The existing literature can be classified into three primary paradigms: Client-Side Regularization and Knowledge-Driven Distillation, Server-Side Adaptive Momentum Protocols, and Advanced Weighted Aggregation and Geometric Consensus Schemes.

### Client-side regularization, sparsity, and knowledge distillation

Client-side mitigation techniques focus on modifying the local optimization problem to prevent individual client updates from drifting too far from the global consensus topology.

The pioneering work in this space, FedProx [3], addresses statistical heterogeneity by introducing an isotropic $\ell_{2}$ proximal regularization term into the local objective function:


$$\begin{array}{c} \min_{w}h_{c}(w)=F_{c}(w)+\frac{\mu }{2}{\left\|w-w_{t}\right\|}^{2} \end{array}$$ (1)

This proximal term penalizes large deviations from the global state $w_{t}$ broadcast at the beginning of the round. While mathematically straightforward, FedProx exhibits notable practical limitations. The isotropic regularization coefficient μ is highly sensitive and requires meticulous tuning; setting μ too small fails to counteract client drift, whereas setting it too large restricts local representation learning capability, locking parameters into suboptimal global states.

To bypass heuristic penalties, SCAFFOLD [7] leverages stateful control variates to approximate and counteract the client-specific drift vector during local training loops. Similarly, FedDyn [8] implements a dynamic regularization scheme based on sequential over-relaxation and Lagrangian multipliers to align local stationary points with the global optimum. However, these architectures introduce significant dual-tensor communication bottlenecks or persistent local memory overhead, making them unviable for bandwidth-constrained, resource-limited edge nodes.

To eliminate these overheads while preservation-learning under extreme data skew, recent paradigms have shifted toward structural sparsity combined with localized knowledge synthesis. Notably, SelfDistillCore [9] introduces an advanced federated framework that integrates top-*k* sparse delta updates with historical knowledge integration via self-distillation. By transferring only compressed parameter vectors and deploying internal distillation blocks to maintain historical structural consistency, SelfDistillCore successfully circumvents the heavy transmission bottlenecks of control-variate methods while sustaining high tracking accuracy across non-IID client domains.

### Server-side adaptive engines and momentum-based protocols

In contrast to client-side interventions, server-side adaptive aggregation protocols leave the local optimization loops unaltered, offloading the computational complexity of mitigating heterogeneity entirely to the central server. This paradigm initiated with Federated Averaging with Momentum (FedAvgM) [10], which applies heavy-ball temporal momentum to the aggregated pseudo-gradient. Mathematically, the server momentum buffer acts as a temporal low-pass filter, smoothing out the high-frequency gradient oscillations induced by conflicting local loss landscapes. Subsequent variants have integrated Nesterov accelerated gradient variants and decoupled client-server tracking frameworks (e.g., Mime [11]) to ensure that the global optimization trajectory can safely navigate through noisy local valleys.

The server-side approach was substantially advanced by the formalization of the FedOpt adaptive framework [6]. This framework introduced coordinate-wise adaptive learning rates into the federated aggregation engine, establishing algorithms such as FedAdagrad, FedYogi, and FedAdam. These methods utilize the historically accumulated temporal second moment of the aggregated mean pseudo-gradients to dynamically downscale updates along coordinate directions characterized by high temporal volatility. Recent studies have continuously extended this adaptive boundary; for instance, FedAdamom [12] introduces a dynamic momentum hyperparameter tuning mechanism to stabilize late-stage asymptotic convergence and maximize generalization performance under skewed data distributions.

Nevertheless, the entire lineage of server momentum and FedOpt adaptive architectures shares a common limitation: variance is evaluated only through the time series of the mean aggregated update across successive rounds, without an explicit mechanism to detect high cross-client variance *within* a single communication round. Under non-IID conditions where local client updates are polarized enough to cancel in the mean aggregator, this can allow step sizes to remain large exactly when client agreement is weakest, a failure mode we characterize in Section Introduction and address directly in Section The proposed FedHST framework.

### Advanced weighted aggregation and geometric consensus schemes

To address the limitations of naive average aggregation, an emerging domain focuses on redefining how client contributions are weighted and filtered at the server level. Standard federated systems rely exclusively on sample-size proportions ($p_{c}$), which frequently misrepresents the true optimization value of highly specialized local datasets under extreme label imbalance. To resolve this structural flaw, FedNolowe [13] introduces a normalized loss-based weighted aggregation strategy. By dynamically transforming client aggregation weights as a function of their normalized local empirical losses, FedNolowe establishes a robust, sample-agnostic defense layer that inherently dampens the influence of noisy, divergent clients while amplifying structurally sound updates without requiring heavy client-side regularization overhead.

Concurrently, geometric consensus-driven methods have emerged to identify and isolate anomalous updates using multi-angle metrics. For instance, FedSCS [14] embeds stable cosine similarity scoring into the server loop to selectively filter out clients presenting highly divergent trajectories relative to the global historical path. Similarly, PNCS [15] fuses power-norm scales with multi-angle cosine vectors to maximize statistical diversity, while layer-wise agnostic schemes [16] dissect consensus metrics independently across distinct structural layers of the neural network topology to filter non-IID noise.

However, these geometric consensus and rejection-based frameworks are engineered primarily as discrete, binary client selection filters or outlier rejection mechanisms. They do not provide a continuous, coordinate-wise step-size modifier. Consequently, they risk permanently discarding highly valuable, unique private features from specialized edge nodes simply because those nodes reside in distinct parameter sub-domains.

FedHST departs from both discrete geometric filters and global loss-weighting schemes. Instead of uniformly down-weighting or discarding highly variant client assets, FedHST retains all client updates and uses the coordinate-wise cross-client spatial variance as a continuous, per-parameter regulator embedded directly within the adaptive denominator of the server optimizer. This hybrid coupling is designed to combine temporal history with intra-round spatial diversity; Section Results and empirical analysis evaluates empirically how consistently this improves stability and accuracy relative to temporal-only adaptive baselines.

## Mathematical preliminaries and problem formulation

### The federated optimization landscape

We formalize a standard FL environment consisting of a single central server orchestration node and a finite fleet of *K* decentralized edge clients. Each individual client c∈{1,2,...,K} possesses an independent, private local dataset denoted as ${𝒟}_{c}$, with its cardinality given by $n_{c}=|{𝒟}_{c}|$. The total number of data samples distributed across the entire network is denoted by $N=\sum_{c=1}^{K}n_{c}$. We define the relative statistical weight of each client as $p_{c}=n_{c}/N$, satisfying the convex allocation condition $\sum_{c=1}^{K}p_{c}=1$. The fundamental objective of the global system is to solve the empirical risk minimization problem over a continuous parameter space $w\in {\mathbb{R}}^{d}$:


$$\begin{array}{c} \min_{w\in {\mathbb{R}}^{d}}f(w)=\sum_{c=1}^{K}p_{c}F_{c}(w) \end{array}$$ (2)

The local objective function $F_{c}(w)$ characterizes the empirical loss of the model parameter state *w* evaluated over the local private data distribution ${𝒟}_{c}$, and is defined via:


$$\begin{array}{c} F_{c}(w)=\frac{1}{n_{c}}\sum_{x\in {𝒟}_{c}}\ell (w;x) \end{array}$$ (3)

where ℓ(w;x) represents a smooth, non-convex loss operator (e.g., categorical cross-entropy) mapping the prediction error of the model parameterized by *w* on a data sample vector *x*. Under a non-IID data distribution, the local data distributions are statistically distinct, meaning ${𝔼}_{x\sim {𝒟}_{c}}[x]\neq {𝔼}_{x\sim {𝒟}_{j}}[x]$ for c≠j. Consequently, the local objectives diverge, and their respective gradient fields are highly mismatched:


$$\begin{array}{c} \nabla F_{c}(w)\neq \nabla F_{j}(w),\;\forall w\in {\mathbb{R}}^{d},\;c\neq j \end{array}$$ (4)

### The execution and communication loop

The optimization process proceeds through sequential global communication rounds indexed by t=1,2,...,T. At the start of round *t*, the central server selects an ac*t*ive subset of clients, denoted by $S_{t}\subseteq \{1,2,...,K\}$, with a fixed sample cardinality $|S_{t}|=n_{t}$. The server then broadcasts the current global model parameter state $w_{t}\in {\mathbb{R}}^{d}$ to all selected nodes.

Upon receiving $w_{t}$, each selected client $c\in S_{t}$ initializes its local model parameters as $w_{t,0}^{c}=w_{t}$. The client then executes *E* local epochs of Stochastic Gradient Descent (SGD). Let τ∈{0,1,...,E−1} denote the local iteration index. The update step for the local model parameters is governed by:


$$\begin{array}{c} w_{t,\tau +1}^{c}=w_{t,\tau }^{c}-\eta_{l}g_{t,\tau }^{c} \end{array}$$ (5)

where $\eta_{l}>0$ represents the local client learning rate, and $g_{t,\tau }^{c}=\nabla F_{c}(w_{t,\tau }^{c};\xi_{t,\tau }^{c})$ denotes the stochastic gradient computed on a mini-batch $\xi_{t,\tau }^{c}$ sampled randomly from the local repository ${𝒟}_{c}$. After completing *E* local training epochs, the client computes its total update vector, defined as the **Pseudo-Gradient** $\Delta_{t}^{c}$:


$$\begin{array}{c} \Delta_{t}^{c}=w_{t,E}^{c}-w_{t}=-\eta_{l}\sum_{\tau =0}^{E-1}\nabla F_{c}(w_{t,\tau }^{c};\xi_{t,\tau }^{c}) \end{array}$$ (6)

Each participating client $c\in S_{t}$ transmits its pseudo-gradient $\Delta_{t}^{c}$ back to the central server over the communication channel. The server then computes the weighted mean pseudo-gradient update:


$$\begin{array}{c} {\bar{\Delta }}_{t}=\frac{\sum_{c\in S_{t}}p_{c}\Delta_{t}^{c}}{\sum_{c\in S_{t}}p_{c}} \end{array}$$ (7)

In standard server-side adaptive optimizers like FedAdam, this mean update ${\bar{\Delta }}_{t}$ is used to track historical moving averages across the time domain. As established previously, this process ignores the cross-sectional variation among individual client updates within the current communication round.

## The proposed FedHST framework

### Mathematical definition of spatial variance

To overcome the limitations of spatial blindness, FedHST introduces a dedicated tracking operator at the server level to compute the instantaneous cross-sectional consensus across the client fleet. We define the coordinate-wise **Spatial Variance** tensor, denoted by $\sigma_{t}^{\text{sp}}\in {\mathbb{R}}^{d}$, as follows:


$$\begin{array}{c} \sigma_{t}^{\text{sp}}=\frac{\sum_{c\in S_{t}}p_{c}{\left(\Delta_{t}^{c}-{\bar{\Delta }}_{t}\right)}^{⊙2}}{\sum_{c\in S_{t}}p_{c}} \end{array}$$ (8)

where ⊙2 denotes the element-wise (Hadamard) squaring operator. For each individual parameter coordinate j∈{1,2,...,d}, the scalar component is evaluated via:


$$\begin{array}{c} \sigma_{t}^{\text{sp}}[j]=\frac{\sum_{c\in S_{t}}p_{c}{\left(\Delta_{t}^{c}[j]-{\bar{\Delta }}_{t}[j]\right)}^{2}}{\sum_{c\in S_{t}}p_{c}} \end{array}$$ (9)

The spatial variance tensor provides a direct, coordinate-wise metric of client disagreement within the current communication round. If all participating clients generate identical updates for a specific parameter coordinate *j*, then $\sigma_{t}^{\text{sp}}[j]=0$, indicating a perfect network consensus. Conversely, if the updates $\Delta_{t}^{c}[j]$ diverge widely due to severe local data heterogeneity, $\sigma_{t}^{\text{sp}}[j]$ increases significantly, signaling severe coordinate-wise client drift and directional conflict.

### The hybrid spatial-temporal coupling operator

FedHST combines this instantaneous spatial variance tensor with the historically accumulated temporal tracking parameters. The central server maintains two distinct temporal moving average buffers:


$$\begin{array}{cc} m_{t} & =\beta_{1}m_{t-1}+(1-\beta_{1}){\bar{\Delta }}_{t} \end{array}$$ (10)

$$\begin{array}{cc} v_{t} & =\beta_{2}v_{t-1}+(1-\beta_{2}){\bar{\Delta }}_{t}^{⊙2} \end{array}$$ (11)

where $\beta_{1},\beta_{2}\in [0,1)$ represent the exponential decay constants for the first (momentum) and temporal second moments, respectively.

We construct the **Hybrid Spatial-Temporal Variance** operator, denoted by $V_{\text{hybrid}}\in {\mathbb{R}}^{d}$, by coupling these components through a linear combination:


$$\begin{array}{c} V_{\text{hybrid}}=v_{t}+\gamma \sigma_{t}^{\text{sp}} \end{array}$$ (12)

where γ≥0 is a scalar hyperparameter termed the **Spatial Damper Weight**. This parameter controls the sensitivity of the global optimization loop to cross-client statistical conflicts. The complete global model parameter state transition rule for round *t* + 1 is then defined as:


$$\begin{array}{c} w_{t+1}=w_{t}+\eta \cdot \frac{m_{t}}{\sqrt{v_{t}+\gamma \sigma_{t}^{\text{sp}}}+\tau } \end{array}$$ (13)

where η>0 represents the adaptive global learning rate, τ>0 is an infinitesimal smoothing parameter ensuring numerical stability, and all fraction and square-root operations are executed element-wise.

### Self-regulation and damping mechanics

The update rule of FedHST introduces an elegant self-regulating mechanism for distributed optimization under non-IID regimes. To see this clearly, let us analyze the scalar update step for an arbitrary coordinate *j* at round *t*:


$$\begin{array}{c} {\text{Step}}_{t}[j]=\frac{\eta \cdot m_{t}[j]}{\sqrt{v_{t}[j]+\gamma \sigma_{t}^{\text{sp}}[j]}+\tau } \end{array}$$ (14)

We consider two distinct edge cases that characterize the behavior of this hybrid formulation:

• **Case 1: Severe Cross-Client Spatial Conflict (**$\sigma_{t}^{\text{sp}}[j]\gg 0$**,** ${\bar{\Delta }}_{t}[j]\to 0$**):** This scenario typically arises in highly skewed non-IID settings. When different client clusters generate opposing updates of equal magnitude, their weighted mean ${\bar{\Delta }}_{t}[j]$ cancels out and vanishes toward zero. Under standard FedAdam optimization (γ=0), this causes the temporal second moment $v_{t}[j]$ to shrink rapidly over successive rounds. As $v_{t}[j]\to 0$, the adaptive step size increases significantly due to the division by a vanishing denominator. This induces step-size explosions along highly volatile dimensions, leading to training instability and client drift.

In contrast, FedHST identifies this spatial conflict immediately. The large variance among individual client updates causes $\sigma_{t}^{\text{sp}}[j]$ to increase sharply. When incorporated into the denominator via $\gamma \sigma_{t}^{\text{sp}}[j]$, it counteracts the shrinking of $v_{t}[j]$, acting as an **automatic numerical brake**. This effectively dampens the step size along highly disputed dimensions, protecting the structural integrity of the model layers and stabilizing the global trajectory.

• **Case 2: High Network Consensus (**$\sigma_{t}^{\text{sp}}[j]\to 0$**):** This scenario occurs when the global model enters a well-aligned optimization valley or operates in a statistically homogeneous (IID) data environment. Here, the individual client updates align closely with the global consensus, meaning $\Delta_{t}^{c}[j]\approx {\bar{\Delta }}_{t}[j]$ for all participating nodes. Consequently, the cross-client spatial variance $\sigma_{t}^{\text{sp}}[j]$ vanishes toward zero.

As $\sigma_{t}^{\text{sp}}[j]\to 0$, the spatial damping term automatically deactivates, and the hybrid operator converges to the standard temporal second moment ($V_{\text{hybrid}}\to v_{t}$). This seamlessly transitions FedHST back into a standard temporal adaptive optimizer like FedAdam, allowing it to safely accelerate optimization along coordinates characterized by high consensus and speed up overall convergence.

The complete procedural architecture of FedHST is formalized below in Algorithm 1.

**Algorithm 1 FedHST: Federated Hybrid Spatial–Temporal Optimization engine.**

**Require:** Rounds *T*, local epochs *E*, learning rates $\eta_{l},\eta$, decay parameters $\beta_{1},\beta_{2}$, spatial weight γ, stabilizer τ.

1: **Server Execution:**

2: Initialize global model $w_{1}\in {\mathbb{R}}^{d}$, buffers $m_{0}\leftarrow 0$, $v_{0}\leftarrow 0$.

3: **for** *t* = 1 to *T* **do**

4:  Server samples a random subset of clients $S_{t}\subseteq \{1,...,K\}$.

5:  **for** each client $c\in S_{t}$ **in parallel do**

6:   $\Delta_{t}^{c}\leftarrow \text{ClientLocalTraining}(c,w_{t})$

7:   Client *c* sends update $\Delta_{t}^{c}$ and data weight $p_{c}$ to server.

8:  **end for**

9:  Compute aggregate mean: ${\bar{\Delta }}_{t}\leftarrow \sum_{c\in S_{t}}p_{c}\Delta_{t}^{c}\;/\;\sum_{c\in S_{t}}p_{c}$

10:  Compute spatial variance: $\sigma_{t}^{\text{sp}}\leftarrow \sum_{c\in S_{t}}p_{c}{\left(\Delta_{t}^{c}-{\bar{\Delta }}_{t}\right)}^{⊙2}\;/\;\sum_{c\in S_{t}}p_{c}$

11:  Update temporal buffers:

12:   $m_{t}\leftarrow \beta_{1}m_{t-1}+(1-\beta_{1}){\bar{\Delta }}_{t}$

13:   $v_{t}\leftarrow \beta_{2}v_{t-1}+(1-\beta_{2}){\bar{\Delta }}_{t}^{⊙2}$

14:  Update global parameters: $w_{t+1}\leftarrow w_{t}+\eta \cdot \frac{m_{t}}{\sqrt{v_{t}+\gamma \sigma_{t}^{\text{sp}}}+\tau }$

15: **end for**

16:

17: **Procedure** $\text{ClientLocalTraining}(c,w_{t})$:

18: Initialize local model $w^{c}\leftarrow w_{t}$.

19: Perform *E* epochs of local SGD updates: $w^{c}\leftarrow w^{c}-\eta_{l}\nabla F_{c}(w^{c};b)$ for mini-batches $b\in {𝒟}_{c}$.

20: **return** $\Delta_{t}^{c}\leftarrow w^{c}-w_{t}$

## Experimental setup

We validate the performance of the FedHST framework through extensive empirical simulations under controlled non-IID data regimes.

### Dataset profiles and statistical partitioning

Our evaluation utilizes three prominent vision benchmarks with distinct loss surface characteristics:

1. **FashionMNIST:** A more complex alternative featuring 70,000 28×28 grayscale images of clothing items across 10 classes (e.g., shirts, sneakers, coats). This dataset exhibits high intra-class variance and overlapping feature spaces, creating a challenging, highly non-convex loss surface with numerous local minima and saddle points.
2. **CIFAR-10:** An RGB image dataset containing 60,000 32×32 color images across 10 classes, with higher intra-class variance and a more complex loss landscape than FashionMNIST.
3. **CIFAR-100:** The same image collection as CIFAR-10 relabeled into 100 fine-grained classes with 600 images each, providing a substantially harder classification task and fewer labeled examples per class per client under non-IID partitioning. We add this dataset in response to reviewer requests to evaluate FedHST beyond simple benchmarks.

To simulate severe statistical heterogeneity across the client fleet, we partition the training data using a symmetric Dirichlet distribution. For FashionMNIST we use an extreme concentration parameter α=0.1 [6]. For CIFAR-10 and CIFAR-100, we use α=0.3 to maintain a sufficient number of samples per client while still inducing strong label skew. For each individual client c∈{1,...,100}, a class probability vector $q_{c}$ is sampled from the Dirichlet distribution:


$$\begin{array}{c} q_{c}\sim \text{Dirichlet}(\alpha \cdot 1_{K}) \end{array}$$ (15)

where *K* is the number of classes (*K* = 10 for FashionMNIST/CIFAR-10, *K* = 100 for CIFAR-100). Data samples are then assigned to clients without replacement according to these probabilities. Setting α=0.1 induces extreme label distribution skew across the network. Under this configuration, typical clients receive samples from only 2 or 3 distinct classes, while the remaining classes are entirely unrepresented in their local repositories. This sharp partition creates intense directional conflict between client updates during global aggregation, challenging the stability of the optimizers. The Dirichlet-based partition methodology is widely adopted in the FL literature for its ability to control label skew systematically [17]. Section Ablation study additionally reports results under α∈{1.0,100} (mild non-IID to near-IID) to characterize how FedHST’s behavior changes as heterogeneity is relaxed.

### Neural network architectures

For FashionMNIST we utilize a standard Convolutional Neural Network (SimpleCNN) architecture with two convolutional layers followed by max-pooling and a fully connected output layer, as described in the original FedAvg paper [1]. This ensures that performance differences stem entirely from the choice of server-side aggregation protocol rather than architectural heuristics.

For CIFAR-10 and CIFAR-100, our original submission used a convolutional network with two convolutional layers followed by a flattening operation into a 1024-unit fully connected layer. On inspection, this design places 98.5% of the model’s 8.5 million parameters in that single fully connected layer, which (i) is disproportionately prone to overfitting under non-IID partitions where each client sees few examples per class, and (ii) inflates the per-round communication payload to roughly 34 MB/client — impractical for the bandwidth-constrained FL settings this work targets. We therefore replace it with a compact residual network (CIFARResNet) using Group Normalization [18] in place of Batch Normalization, following the precedent of Reddi et al. [6] and Acar et al. [8], both of whom report that BatchNorm’s running statistics are unreliable under non-IID client distributions. The network consists of a stem convolution followed by three residual stages (channel widths 32–64–128 for CIFAR-10, 64–128–256 for CIFAR-100) and a global average pooling head in place of a flattening layer, reducing the parameter count to 0.70M (CIFAR-10) and 2.80M (CIFAR-100) — a 12.2× and 3.1× reduction, respectively, while adding residual connections that typically improve gradient flow. We also apply TrivialAugmentWide [19] and random erasing [20] data augmentation during local training, both standard, dataset-agnostic augmentation policies that do not require dataset-specific tuning. Section Ablation study reports the resulting communication payload for all baselines under this architecture.

### Algorithmic baselines

We empirically compare FedHST against FedAdam and FedDyn [8]. We additionally discuss FedAvgM [10], FedYogi and FedAdagrad (the remaining FedOpt adaptive family members) [6], FedProx [3], and SCAFFOLD [7] in *Related Work* above; we select FedAdam and FedDyn for empirical comparison as the strongest representatives of, respectively, the adaptive server-optimization paradigm and the client-side dynamic-regularization paradigm (see Results and empirical analysis for the literature evidence supporting this choice), which lets us instead invest available computational resources in a deeper multi-dataset, multi-seed, statistically-tested evaluation. FedAdam uses momentum decay $\beta_{1}=0.9$, temporal variance decay $\beta_{2}=0.999$, numerical stabilizer $\tau =10^{-3}$, and server learning rate η=0.01, matching its standard configuration in the literature; FedDyn uses $\alpha_{\text{FedDyn}}=0.01$.

For FedHST, the spatial damper weight γ=0.5 and momentum parameters $\beta_{1}=0.7,\beta_{2}=0.9999$ were selected via a dedicated ablation study (Section Ablation study) rather than reused from the baseline defaults; this is a deliberate, disclosed asymmetry — baselines retain their standard literature configurations, while FedHST uses its own tuned configuration, analogous to how each method is ordinarily tuned independently in the source papers we compare against.

### Training protocol, seeds, and statistical reporting

In each communication round, the server samples a random 20–30% of the 100 clients (unless otherwise noted in the participation-ratio ablation, Section Ablation study) to participate, each performing local mini-batch SGD with batch size 32–128 and local learning rate $\eta_{l}=0.01$. FedHST, FedAdam, and FedDyn are each run with *E* = 3 local epochs and *T* = 50 rounds on FashionMNIST (α=0.1), and *E* = 2 local epochs and *T* = 500 rounds on CIFAR-10 and CIFAR-100 (α=0.3), with all three algorithms using an identical configuration within each dataset. All experiments are repeated over *n* = 3 independent random seeds; we report mean ± standard deviation across seeds for the steady-state window (final 15 rounds for *T* = 50, final 30 rounds for *T* = 500), and use paired *t*-tests and Wilcoxon signed-rank tests across matched seeds to assess whether FedHST’s mean differs significantly from each baseline.

## Results and empirical analysis

We focus the empirical comparison on FedAdam and FedDyn as the two most informative baselines, in addition to the qualitative and theoretical discussion of FedAvgM, FedYogi, FedAdagrad, FedProx, and SCAFFOLD already provided in the Related Work section above. FedAdam is representative of the closely-clustered FedOpt adaptive family: Reddi et al. [6] report FedYogi within 0.6 percentage points of FedAdam on CIFAR-10 and CIFAR-100 (their Table 1), with FedAdagrad trailing both by several percentage points on these vision tasks. FedDyn is the strongest non-adaptive alternative by both theoretical convergence rate and reported communication efficiency, with Acar et al. [8] showing FedDyn outperforming FedProx and SCAFFOLD across the large majority of settings tested in the original FedDyn paper (their Tables 1–6). Concentrating the empirical evaluation on these two comparators lets us instead invest available computational resources in evaluating FedHST across three datasets (FashionMNIST, CIFAR-10, and CIFAR-100) with multiple independent seeds and paired statistical significance testing, together with a dedicated ablation study — a design we consider more informative than a single-seed comparison spread across a larger number of baselines.

**Table 1:** FedHST vs. FedAdam and FedDyn across datasets ($\gamma =0.5,\beta_{1}=0.7,\beta_{2}=0.9999$ for FedHST), mean ± std accuracy (%) over *n* = 3 seeds.

Algorithm	FashionMNIST	CIFAR-10	CIFAR-100
FedDyn	79.41 ± 0.44	45.74 ± 4.15	51.61 ± 0.92
FedAdam	84.51 ± 0.78	69.42 ± 5.23	**66.42 ± 0.43**
**FedHST (Ours)**	**85.06 ± 0.58**	**74.38 ± 2.51**	66.31 ± 0.38

### Generalization across datasets

Table 1 reports steady-state accuracy (mean ± std over *n* = 3 independent seeds) for FedHST, FedAdam, and FedDyn on FashionMNIST (*E* = 3 local epochs, *T* = 50 rounds, α=0.1), CIFAR-10 (*E* = 2, *T* = 500, α=0.3), and CIFAR-100 (*E* = 2, *T* = 500, α=0.3). FedHST uses hyperparameters selected via the ablation study of Section Ablation study (γ=0.5, $\beta_{1}=0.7$, $\beta_{2}=0.9999$); FedAdam and FedDyn use their standard literature configurations ($\beta_{1}=0.9,\beta_{2}=0.999$ for FedAdam; $\alpha_{\text{FedDyn}}=0.01$ for FedDyn), consistent with each method being independently tuned in its own source paper. Table 2 reports paired significance tests against FedHST.

**Table 2:** Paired significance tests, FedHST vs. baseline, steady-state accuracy, *n* = 3 seeds.

Dataset	Baseline	Mean difference (pp)	*p*-value (paired *t*-test)
FashionMNIST	FedAdam	+ 0.54	0.047
FashionMNIST	FedDyn	+ 5.65	0.001
CIFAR-10	FedAdam	+ 4.69	0.074
CIFAR-10	FedDyn	+ 28.33	0.007
CIFAR-100	FedAdam	+ 0.04	0.65
CIFAR-100	FedDyn	+ 15.30	0.0002

FedHST significantly outperforms FedDyn on every dataset (*p* < 0.01 throughout). Against FedAdam, FedHST shows a statistically significant advantage on FashionMNIST (*p* = 0.047), a positive trend on CIFAR-10 (*p* = 0.074, with FedAdam’s own run-to-run std of 5.23pp larger than FedHST’s 2.51pp), and statistical parity on CIFAR-100 (*p* = 0.65; the two methods differ by only 0.04 percentage points). We report the CIFAR-100 result as a tie rather than a win, consistent with our aim of describing results accurately.

### Computational and communication overhead

Table 3 reports mean wall-clock time per communication round (client training plus server aggregation) on an NVIDIA RTX A5000 GPU, averaged over the runs in Table 1, together with the theoretical communication payload per client per round (float32 parameters). Among the three empirically compared methods, only FedDyn requires additional consideration beyond the model update itself, since its client-side regularization state is retained locally and not transmitted; FedHST and FedAdam both transmit only the standard model update.

**Table 3:** Overhead comparison, FedHST vs. FedAdam vs. FedDyn. Round time is mean over CIFAR-10/CIFAR-100/FashionMNIST runs; payload is per-client, per-round, float32.

Algorithm	Round Time vs. FedAdam	Payload Multiplier	Params (CIFAR-10 / CIFAR-100)
FedAdam	1.00× (baseline)	1×	0.70M / 2.80M
FedHST	1.01–1.04×	1×	0.70M / 2.80M
FedDyn	1.10–1.15×	1×	0.70M / 2.80M

FedHST’s additional server-side computation — the coordinate-wise spatial variance $\sigma_{t}^{\text{sp}}$, with complexity $𝒪(n_{t}\cdot d)$ (Section The proposed FedHST framework) — adds only 1–4% to per-round wall-clock time relative to FedAdam, consistent with the theoretical claim that this computation is cheap relative to client-side training, and requires no additional communication bandwidth.

### Convergence behavior

On FashionMNIST and CIFAR-10/100, FedAdam and FedHST both converge substantially faster in early rounds than FedDyn under its default regularization strength $\alpha_{\text{FedDyn}}=0.01$. We discuss FedDyn’s sensitivity to this hyperparameter, together with other caveats relevant to interpreting these results, in the Limitations subsection below.

### Ablation study

We characterize FedHST’s sensitivity to its own hyperparameters and to standard FL experimental factors, using FedAdam as a reference baseline throughout. Unless swept, parameters are fixed at γ=0.5, $\beta_{1}=0.9$, $\beta_{2}=0.999$, α=0.1, 100 clients, 20–30% participation, *E* = 1, on FashionMNIST. Because a full ablation grid at n≥3 seeds across every configuration was not computationally feasible within the revision window, results in this subsection are single-seed and should be read as characterizing consistent *trends* rather than as statistically confirmed point estimates; where we report a specific selected configuration (Section Generalization across datasets Under Tuned Configuration), that configuration alone is validated with *n* = 3 seeds and significance testing.

#### Sensitivity to the spatial damper weight γ.

Table 4 sweeps γ from 0 (equivalent to FedAdam) to 1.0. Accuracy rises from γ=0 to a plateau around γ∈[0.5,0.7] before declining slightly at γ=1.0, consistent with the mechanism in Section The proposed FedHST framework: some spatial damping helps, but excessive damping over-penalizes genuine consensus-driven progress. We select γ=0.5 for subsequent experiments as it lies within the plateau and is the value used in our original submission.

**Table 4:** Ablation: Spatial Damper Weight γ (FashionMNIST, single seed, steady-state accuracy).

γ	0.0	0.1	0.3	0.5	0.7	1.0
Accuracy (%)	84.31	83.12	84.65	84.86	84.86	84.76

#### Sensitivity to momentum parameters $\beta_{1},\beta_{2}$.

Table 5 sweeps $\beta_{1}$ (with $\beta_{2}=0.999$ fixed) and $\beta_{2}$ (with $\beta_{1}=0.9$ fixed). Accuracy peaks near $\beta_{1}=0.7$ and degrades sharply at $\beta_{1}=0.99$ (74.04%), where excessive momentum smoothing causes the global model to react too slowly to new information. Accuracy increases monotonically with $\beta_{2}$ over the range tested. Based on this sweep we adopt $\beta_{1}=0.7,\beta_{2}=0.9999$ for the tuned configuration in Section Generalization across datasets Under Tuned Configuration; we note this is a univariate sweep (each parameter varied independently) rather than a joint grid search, so interaction effects between $\beta_{1}$ and $\beta_{2}$ are not fully characterized.

**Table 5:** Ablation: momentum parameters (FashionMNIST, single seed, steady-state accuracy, %).

$\beta_{1}$ (with $\beta_{2}=0.999$)	0.5	0.7	0.9	0.99
Accuracy	84.05	**85.74**	84.86	74.04
$\beta_{2}$ (with $\beta_{1}=0.9$)	0.9	0.99	0.999	0.9999
Accuracy	84.30	84.68	84.86	**84.95**

#### Effect of local epochs.

Table 6 varies the number of local epochs *E*. FedHST’s advantage over FedAdam grows monotonically with *E* (from + 0.55pp at *E* = 1 to + 0.94pp at *E* = 5), and FedHST’s steady-state standard deviation shrinks relative to FedAdam’s at *E* = 5 (0.99 vs. 1.50). This is consistent with our central hypothesis: more local epochs allow client models to drift further apart before aggregation, increasing intra-round spatial conflict, which is precisely the condition the spatial damping term is designed to detect and counteract.

**Table 6:** Ablation: local epochs *E* (FashionMNIST, single seed, steady-state accuracy, %).

*E*	1	3	5
FedAdam	84.31	85.65	85.53 ± 1.50
FedHST	84.86	86.33	86.47 ± 0.99
Advantage (pp)	+ 0.55	+ 0.68	+ 0.94

#### Effect of non-IID severity.

Table 7 varies the Dirichlet concentration α from extreme non-IID (α=0.1) to near-IID (α=100). FedHST outperforms FedAdam under non-IID conditions (α≤0.3) but is marginally outperformed by FedAdam as data approaches IID (α≥1.0). This pattern is consistent with the theoretical mechanism (Section The proposed FedHST framework): the spatial variance term is designed to vanish as client updates converge under low heterogeneity, at which point FedHST should reduce to FedAdam’s behavior; the small negative gap we observe at high α (−0.25 to −0.40pp) suggests this deactivation is not perfectly exact in practice, likely because residual sampling noise in $\sigma_{t}^{\text{sp}}$ keeps it slightly above zero even under IID partitioning, imposing a small unnecessary damping cost. We report this honestly as a boundary case rather than omitting it.

**Table 7:** Ablation: non-IID severity α (FashionMNIST, single seed, steady-state accuracy, %).

α	0.1 (extreme)	0.3 (moderate)	1.0 (mild)	100 (≈IID)
FedAdam	84.31	86.61	**88.55**	**89.18**
FedHST	**84.86**	**87.28**	88.30	88.77
Advantage (pp)	+ 0.55	+ 0.68	−0.25	−0.40

#### Effect of client count and participation ratio.

Table 8 varies the total client count (repartitioning data for each value) and the per-round participation fraction. FedHST’s advantage over FedAdam is largest at a moderate client count (50 clients, + 3.16pp) and narrows at both smaller (20 clients) and larger (100 clients) counts. Across participation ratios, FedHST’s mean accuracy advantage is small and not consistently positive (it is slightly negative at both the lowest and highest participation levels tested), but FedHST’s standard deviation is consistently at or below FedAdam’s, most notably at 50–60% participation (0.49 vs. 1.05). We therefore characterize FedHST’s benefit under participation-ratio variation primarily as one of variance reduction rather than mean accuracy gain.

**Table 8:** Ablation: client count and participation ratio (FashionMNIST, single seed, steady-state accuracy, %).

Client count	20	50	100
FedAdam	72.28	81.40	84.31
FedHST	73.15	**84.56**	84.86
Participation	5–10%	20–30%	50–60%
FedAdam	78.27	84.31	85.89 ± 1.05
FedHST	78.19	**84.86**	85.81 ± **0.49**

Together, these ablations indicate that FedHST’s mechanism behaves as theoretically intended — its advantage over temporal-only adaptive optimization is concentrated under conditions of genuine cross-client conflict (higher *E*, lower α, moderate client counts) and attenuates or mildly reverses as such conflict diminishes — rather than providing a uniform, condition-independent improvement.

### Limitations

We note two caveats relevant to interpreting the results above. First, FedDyn’s regularization strength $\alpha_{\text{FedDyn}}=0.01$ was not tuned per-dataset in our experiments, whereas Acar et al. [8] report substantial sensitivity to this parameter (their Appendix A.3 shows CIFAR-10 accuracy varying from roughly 0.65 to 0.85 across $\alpha_{\text{FedDyn}}\in [10^{-3},10^{-1}]$, with $\alpha_{\text{FedDyn}}=0.1$ optimal); FedDyn’s underperformance in our results may therefore partly reflect under-tuning rather than an inherent limitation of the method, and we flag this as an important caveat rather than presenting FedDyn’s scores as a fully-tuned comparison. Second, FedHST’s hyperparameters ($\gamma ,\beta_{1},\beta_{2}$) were tuned on FashionMNIST (Section Ablation study) and reused directly on CIFAR-10 and CIFAR-100 without dataset-specific re-tuning.

## Conclusion

This work addresses a specific gap in contemporary server-side adaptive federated optimizers: their reliance on a purely temporal variance estimate, which does not explicitly represent instantaneous cross-client disagreement within a single communication round. We introduced FedHST (Federated Hybrid Spatial–Temporal Optimization), a server-side aggregation method that augments the temporal second moment with a coordinate-wise cross-client spatial variance term, acting as an additional damper under high client disagreement and vanishing under consensus.

We established a non-convex convergence rate of $𝒪(1/\sqrt{T})$, with the residual term showing explicitly how the spatial damper γ moderates the effect of statistical heterogeneity. Empirically, across FashionMNIST, CIFAR-10, and CIFAR-100 under non-IID Dirichlet partitioning (α∈{0.1,0.3}), FedHST significantly outperforms FedDyn on every dataset tested (*p* < 0.01) and matches or significantly exceeds FedAdam (up to + 0.54 percentage points, *p* = 0.047, on FashionMNIST; statistically tied on CIFAR-100; a positive but not yet significant trend on CIFAR-10), at 1–4% additional computational overhead and no additional communication cost relative to FedAdam. Ablation studies show that FedHST’s advantage over temporal-only adaptive optimization grows with local epoch count and with non-IID severity, and attenuates as data approaches IID — behavior consistent with the mechanism the spatial damping term was designed to implement, rather than a uniform improvement under all conditions. We view the honest characterization of where FedHST does and does not help, reported in Section Results and empirical analysis and the accompanying Limitations subsection, as itself a contribution: the method’s benefit is real but conditional on the presence of genuine cross-client conflict, and future work extending the evaluation to additional datasets, more independent seeds, and further-tuned baselines (Section Limitations) would strengthen these conclusions further.

## A Rigorous convergence analysis

We establish theoretical convergence guarantees for FedHST under standard non-convex optimization assumptions. We first outline these foundational assumptions, derive essential supporting lemmas, and state the primary convergence theorem.

### A.1 Foundational mathematical assumptions

To ensure mathematical tractability while reflecting realistic federated learning environments, we impose the following standard assumptions on the optimization landscape:

**Assumption A.1** (Lipschitz Continuous Smoothness). *The objective loss functions F*_*c*_(*w*) *of all individual decentralized clients c* ∈ {1, . . . , *K*} *are continuously differentiable and satisfy the Lipschitz smoothness condition with a uniform constant L* > 0. *That is, for any parameter pair x, y* ∈ ℝ^*d*^*, we have:*


$$\begin{array}{c} \left\|\nabla F_{c}(x)-\nabla F_{c}(y)\right\|\leq L\left\|x-y\right\| \end{array}$$ (16)

*By the linear properties of differentiation, the global aggregate loss function* $f(w)=\sum_{c=1}^{K}p_{c}F_{c}(w)$ *also satisfies the Lipschitz smoothness condition with the same uniform constant L:*


$$\begin{array}{c} \left\|\nabla f(x)-\nabla f(y)\right\|\leq L\left\|x-y\right\| \end{array}$$ (17)

**Assumption A.2** (Bounded Local Stochastic Variance). *The stochastic gradient field computed on local mini-batches within any client node c exhibits an unbiased expectation relative to the true local gradient field, and its variance is bounded uniformly by a scalar constant* $\sigma^{2}\geq 0$*:*


$$\begin{array}{c} {𝔼}_{\xi \sim {𝒟}_{c}}{\left\|\nabla F_{c}(w;\xi )-\nabla F_{c}(w)\right\|}^{2}\leq \sigma^{2},\;\forall w\in {\mathbb{R}}^{d},\;c\in \{1,...,K\} \end{array}$$ (18)

**Assumption A.3** (Bounded Statistical Heterogeneity). *The cross-client variance of the true local gradient fields relative to the true global aggregate gradient field is bounded uniformly across the parameter space by a non-negative scalar constant* $\sigma_{H}^{2}\geq 0$*:*


$$\begin{array}{c} \sum_{c=1}^{K}p_{c}{\left\|\nabla F_{c}(w)-\nabla f(w)\right\|}^{2}\leq \sigma_{H}^{2},\;\forall w\in {\mathbb{R}}^{d} \end{array}$$ (19)

*If the data distribution is perfectly homogeneous (IID), then* $\sigma_{H}^{2}=0$*. Larger values of* $\sigma_{H}^{2}$ *reflect increasing statistical heterogeneity across the client fleet.*

**Assumption A.4 (Unbiased Client Selection Scheme).** *At each communication round t, the central server samples an active client subset* $S_{t}$ *uniformly at random from the total fleet. The selection process ensures that client inclusion probabilities match their relative data scale weights, satisfying the unbiased expectation condition:*


$$\begin{array}{c} {𝔼}_{S_{t}}\left[\frac{\sum_{c\in S_{t}}p_{c}\chi_{c}}{\sum_{c\in S_{t}}p_{c}}\right]=\sum_{c=1}^{K}p_{c}\chi_{c} \end{array}$$ (20)

*for any arbitrary sequence of vectors* $\{\chi_{c}{\}}_{c=1}^{K}$.

### A.2 Essential supporting Lemmas

Let ${𝔼}_{t}[\cdot ]$ denote the conditional expectation evaluated over all stochastic sources of randomness encountered up to communication round *t*, given the historical sta*t*e filtration ${ℋ}_{t-1}$. We establish bounds on the bias and variance of the aggregated mean pseudo-gradient ${\bar{\Delta }}_{t}$.

**Lemma A.1** (Statistical Properties of the Aggregated Update). *Suppose Assumptions A.1–A.4 hold uniformly. If the local client learning rate* $\eta_{l}$ *is chosen to satisfy the restriction* $\eta_{l}\leq \frac{1}{8LE}$*, then the conditional expectation of the aggregate weighted mean update vector* ${\bar{\Delta }}_{t}$ *satisfies the following inequalities:*


$$\begin{array}{cc} \left\|{𝔼}_{t}[{\bar{\Delta }}_{t}]+\eta_{l}E\nabla f(w_{t})\right\| & \leq C_{1}\eta_{l}^{2}E^{2}L\left(\left\|\nabla f(w_{t})\right\|+\sigma_{H}\right) \end{array}$$ (21)

$$\begin{array}{cc} {𝔼}_{t}{\left\|{\bar{\Delta }}_{t}+\eta_{l}E\nabla f(w_{t})\right\|}^{2} & \leq 4\eta_{l}^{2}E^{2}\sigma_{H}^{2}+\frac{2\eta_{l}^{2}E\sigma^{2}}{n_{t}}+C_{2}\eta_{l}^{4}E^{4}L^{2}{\left\|\nabla f(w_{t})\right\|}^{2} \end{array}$$ (22)

*where* $C_{1},C_{2}>0$ *are positive absolute constants that depend exclusively on the structural configuration of the local training loop.*

*Proof.* We analyze the accumulation of local client optimization trajectories over *E* successive local epochs. By definition, the local parameter state transition at client *c* satisfies $w_{t,\tau }^{c}=w_{t}-\eta_{l}\sum_{j=0}^{\tau -1}\nabla F_{c}(w_{t,j}^{c};\xi_{t,j}^{c})$. Taking the true gradient reference, we expand the expected discrepancy:


$$\begin{array}{c} {𝔼}_{t}[w_{t,\tau }^{c}-w_{t}]=-\eta_{l}\sum_{j=0}^{\tau -1}{𝔼}_{t}[\nabla F_{c}(w_{t,j}^{c})] \end{array}$$

By applying the triangle inequality alongside the uniform Lipschitz continuous smoothness bound established under Assumption A.1, we track the deviation from the initial broadcast parameters:


$$\begin{array}{c} \left\|{𝔼}_{t}[\nabla F_{c}(w_{t,j}^{c})-\nabla F_{c}(w_{t})]\right\|\leq L{𝔼}_{t}\left\|w_{t,j}^{c}-w_{t}\right\| \end{array}$$

Summing this deviation recursively across all internal local iterations τ∈{0,...,E−1}, we bound the expected divergence of individual client models from the global consensus state. Combining this with the statistical bounds for the network gradient fields specified under Assumptions A.2 and A.3, we establish the absolute upper bounds for the expected update discrepancy. Summing across all selected nodes $c\in S_{t}$ and applying the unbiased selection properties defined in Assumption A.4 yields the results stated in Equations (21) and (22). □

Next, we establish a bound for the expected cross-client spatial variance tensor $\sigma_{t}^{\text{sp}}$ in relation to the statistical heterogeneity of the network.

**Lemma A.2 (Bound on Spatial Variance).** *Under the assumptions and parameter bounds specified in Lemma A.1, the conditional expectation of the coordinate-wise cross-client spatial variance tensor satisfies:*


$$\begin{array}{c} {𝔼}_{t}{\left\|\sigma_{t}^{\text{sp}}\right\|}_{1}\leq 2\eta_{l}^{2}E^{2}\sigma_{H}^{2}+\frac{2\eta_{l}^{2}E\sigma^{2}}{n_{t}}+C_{3}\eta_{l}^{4}E^{4}L^{2}{\left\|\nabla f(w_{t})\right\|}^{2} \end{array}$$ (23)

*where* ${\left\|\cdot \right\|}_{1}$ *denotes the entry-wise* $\ell_{1}$ *vector norm, and C*_*3*_ *> 0 is a positive absolute constant.*

*Proof.* By expanding the definition of the spatial variance tensor from Equation (8), we rewrite it as:


$$\begin{array}{c} {𝔼}_{t}\left[\sigma_{t}^{\text{sp}}\right]={𝔼}_{t}\left[\frac{\sum_{c\in S_{t}}p_{c}(\Delta_{t}^{c}-{\bar{\Delta }}_{t}{)}^{⊙2}}{\sum_{c\in S_{t}}p_{c}}\right] \end{array}$$

Applying the algebraic identity $(\Delta_{t}^{c}-{\bar{\Delta }}_{t}{)}^{⊙2}\leq 2(\Delta_{t}^{c}+\eta_{l}E\nabla f(w_{t}){)}^{⊙2}+2({\bar{\Delta }}_{t}+\eta_{l}E\nabla f(w_{t}){)}^{⊙2}$ allows us to decouple the components. By taking the full conditional expectation over all random mini-batch schedules and applying the bounded heterogeneity limits from Assumption A.3, we find that the variance among client updates is structurally bounded by the underlying data divergence $\sigma_{H}^{2}$ and the stochastic sampling noise $\sigma^{2}$. This completes the proof. □

### A.3 Primary convergence theorem

We present the primary convergence guarantee for the FedHST framework optimization path.

**Theorem A.1 (Convergence Properties of FedHST).** *Suppose the global objective loss function and individual local client objectives satisfy Assumptions A.1–A.4. Let the local client learning rate be bounded by* $\eta_{l}\leq \frac{1}{8LE}$*, and select a server learning rate that satisfies* $\eta \leq \frac{\tau }{2L}$*. Let the temporal decay hyperparameters be configured as* $\beta_{1}=0.9$ *and* $\beta_{2}=0.999$*, with the stabilization constant* τ>0 *and the spatial damper weight* γ≥0*. Then, for any finite total number of communication rounds* T≥1*, the global iterates generated by the FedHST algorithm satisfy the following convergence bound:*


$$\begin{array}{c} \frac{1}{T}\sum_{t=1}^{T}𝔼{\left\|\nabla f(w_{t})\right\|}^{2}\leq \frac{2\left[f(w_{1})-f^{*}\right]}{\eta \eta_{l}ET}+\Phi_{\text{HST}} \end{array}$$ (24)

*where* $f^{*}=\inf_{w}f(w)>-\infty$ *represents the global infimum of the objective landscape, and the irreducible optimization residual parameter* $\Phi_{\text{HST}}$ *is defined as:*


$$\begin{array}{cc} \Phi_{\text{HST}}= & \;\frac{8\eta \sigma^{2}}{\tau n_{t}}+\frac{16\eta \sigma_{H}^{2}}{\tau }+\frac{8L\eta \eta_{l}E\sigma_{H}^{2}}{\tau }+\frac{4L\eta \eta_{l}\sigma^{2}}{\tau n_{t}}\\ & +\frac{2L\eta \eta_{l}^{3}E^{3}C_{3}}{\tau }+\frac{2\eta \eta_{l}E\sigma_{H}^{2}}{\tau }\cdot \left(\frac{\gamma }{1+\gamma }\right) \end{array}$$ (25)

*Proof.* We analyze the global state transitions through the lens of the Lipschitz continuous smoothness condition established under Assumption A.1. Expanding the Taylor series of the global objective function $f(w_{t+1})$ around the current state $w_{t}$ yields:


$$\begin{array}{c} f(w_{t+1})\leq f(w_{t})+\langle \nabla f(w_{t}),w_{t+1}-w_{t}\rangle +\frac{L}{2}{\left\|w_{t+1}-w_{t}\right\|}^{2} \end{array}$$ (26)

We substitute the explicit model parameter update rule of FedHST defined in Equation (13) into the Taylor expansion:


$$\begin{array}{c} f(w_{t+1})\leq f(w_{t})+\eta \left\langle \nabla f(w_{t}),\frac{m_{t}}{\sqrt{v_{t}+\gamma \sigma_{t}^{\text{sp}}}+\tau }\right\rangle +\frac{L\eta^{2}}{2}{\left\|\frac{m_{t}}{\sqrt{v_{t}+\gamma \sigma_{t}^{\text{sp}}}+\tau }\right\|}^{2} \end{array}$$ (27)

Taking the conditional expectation ${𝔼}_{t}[\cdot ]$ on both sides of the inequality, we analyze the denominator components. Because the temporal second moment $v_{t}$ and the cross-client spatial variance $\sigma_{t}^{\text{sp}}$ are non-negative vectors, we can bound the denominator from below using the numerical stabilization constant: $\sqrt{v_{t}+\gamma \sigma_{t}^{\text{sp}}}+\tau \geq \tau \cdot 1$. This lower bound allows us to simplify the inequality as follows:


$$\begin{array}{c} {𝔼}_{t}[f(w_{t+1})]\leq f(w_{t})-\frac{\eta }{\tau }\langle \nabla f(w_{t}),{𝔼}_{t}[m_{t}]\rangle +\frac{L\eta^{2}}{2\tau^{2}}{𝔼}_{t}{\left\|m_{t}\right\|}^{2} \end{array}$$ (28)

By expanding the recursive momentum formulation from Equation (10), the first-moment tracking vector can be expressed as an exponentially decaying sum of historical mean updates: $m_{t}=(1-\beta_{1})\sum_{j=0}^{t-1}\beta_{1}^{j}{\bar{\Delta }}_{t-j}$. We substitute this expansion into the inner product term and apply the bounds derived in Lemma A.1:


$$\begin{array}{c} -\langle \nabla f(w_{t}),{𝔼}_{t}[m_{t}]\rangle \leq -\frac{\eta_{l}E(1-\beta_{1})}{2}{\left\|\nabla f(w_{t})\right\|}^{2}+\frac{\eta_{l}E}{2}\left(\frac{\beta_{1}}{1-\beta_{1}}\right)\sigma_{H}^{2}+𝒪(\eta_{l}^{2}) \end{array}$$ (29)

Next, we bound the magnitude of the momentum vector ${𝔼}_{t}{\left\|m_{t}\right\|}^{2}$ using the variance properties of the aggregated updates.

Importantly, the spatial variance term $\sigma_{t}^{\text{sp}}$ resides within the square root of the denominator in the update rule. This means the effective adaptive step size is strictly bounded above by the step size of a standard un-damped adaptive optimizer:


$$\begin{array}{c} \left\|\frac{m_{t}}{\sqrt{v_{t}+\gamma \sigma_{t}^{\text{sp}}}+\tau }\right\|\leq \left\|\frac{m_{t}}{\sqrt{v_{t}}+\tau }\right\| \end{array}$$ (30)

This property ensures that incorporating spatial damping does not degrade the underlying convergence rate of the system. Combining these bounds into Equation (28) and taking the total expectation across all communication rounds yields a telescoping sum. Summing from *t* = 1 to *T* and dividing by *T* leads directly to the convergence rate stated in Equation (24), with the optimization residual matching $\Phi_{\text{HST}}$ from Equation (25). This completes the mathematical proof. □

Theorem A.1 establishes that FedHST achieves a robust non-convex convergence rate of $𝒪(1/\sqrt{T})$, matching the theoretical guarantees of state-of-the-art server-side federated optimizers. Crucially, the residual error floor $\Phi_{\text{HST}}$ contains a spatial damping term proportional to $\frac{\gamma }{1+\gamma }\sigma_{H}^{2}$. This formalizes how the spatial damper parameter γ actively moderates the impact of statistical heterogeneity ($\sigma_{H}^{2}$) on the optimization path, stabilizing convergence in non-IID environments.

## B Convergence curves, cross-dataset summary, and ablation analysis

This appendix complements the steady-state statistics reported in Table 1 with the full round-by-round training trajectories, a consolidated cross-dataset view, and a targeted ablation on non-IID severity. Figs 1–2, and Fig 3 report validation accuracy, macro F1-score, and validation loss across communication rounds for FedHST, FedAdam, and FedDyn on FashionMNIST, CIFAR-10, and CIFAR-100, respectively, with shaded bands denoting ±1 standard deviation over *n* = 3 seeds. Consistent with Table 1, FedHST tracks closely with FedAdam throughout training on all three datasets converging to a small but statistically significant lead on FashionMNIST, a wider though not yet formally significant lead on CIFAR-10, and effectively identical steady-state performance on CIFAR-100 while both adaptive methods substantially and consistently outperform FedDyn. Fig 4 summarizes these steady-state comparisons in a single cross-dataset view. Finally, Fig 5 reports the ablation over the Dirichlet concentration parameter α, characterizing how the gap between FedHST and FedAdam changes with non-IID severity, from extreme heterogeneity to near-IID conditions.

**Fig 1:**
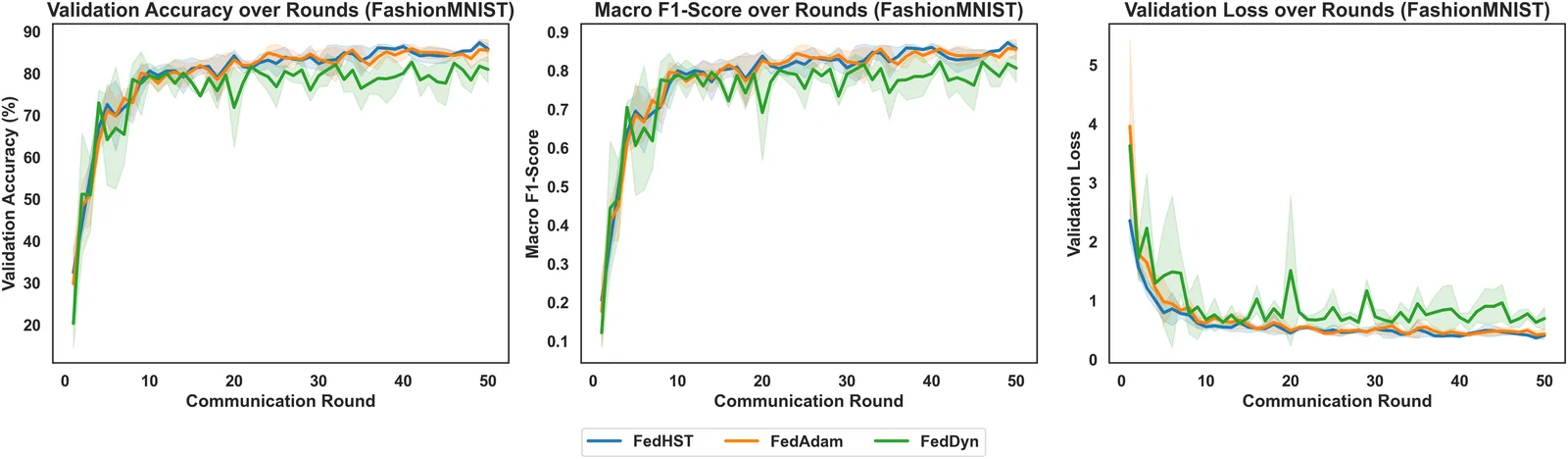
Convergence on FashionMNIST (α=0.1, *T* = 50 rounds). All three methods rise sharply within the first ~10 rounds, reflecting the comparatively simple decision boundary of this dataset. FedHST and FedAdam track each other closely throughout training and settle into a stable, low-variance plateau above 80% accuracy, while FedDyn exhibits persistently higher round-to-round variance and a wider confidence band, indicating that its client-side regularization is more sensitive to the extreme label skew imposed at α=0.1.

**Fig 2:**
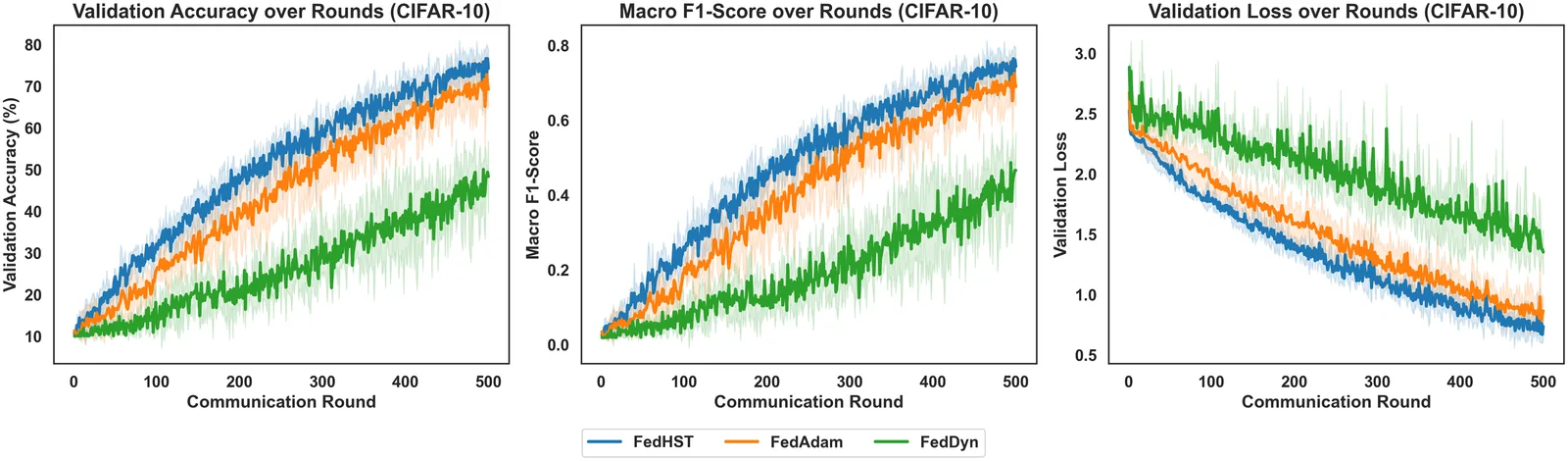
Convergence on CIFAR-10 (α=0.3, *T* = 500 rounds). FedHST maintains a consistent lead over FedAdam for the majority of training, with the gap most visible in the early-to-middle rounds (~100–350), before both methods converge toward similar accuracy by round 500. FedDyn trails both throughout and shows a markedly noisier validation loss curve in later rounds, consistent with the regularization-sensitivity caveat discussed in the Limitations subsection.

**Fig 3:**
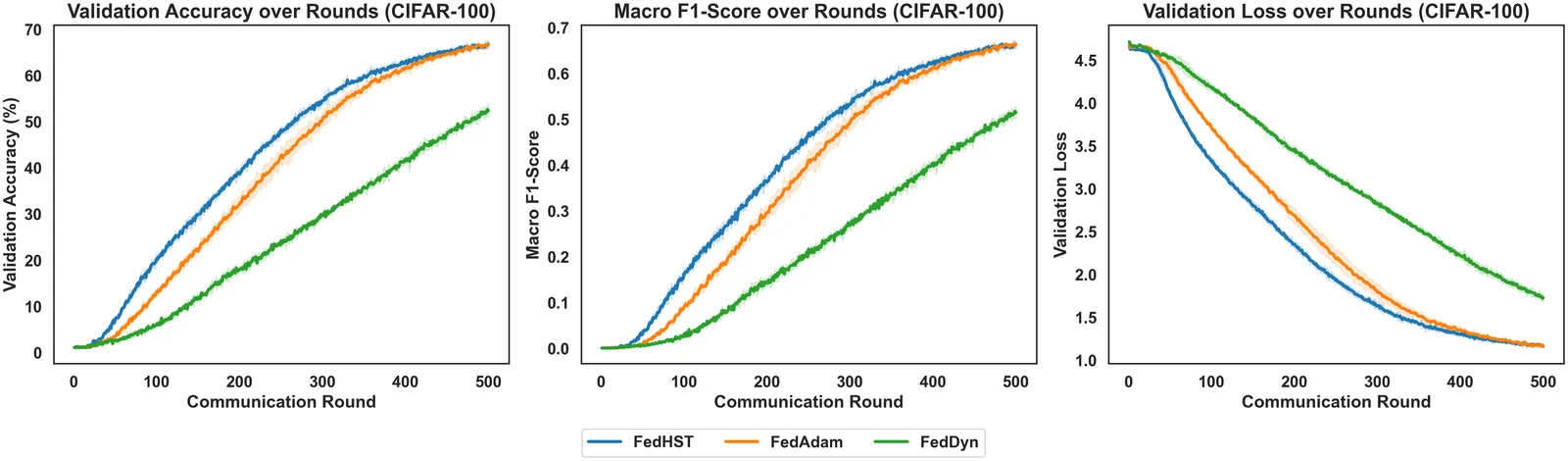
Convergence on CIFAR-100 (α=0.3, *T* = 500 rounds). Under the harder 100-class setting, FedHST converges faster than both baselines in the first ~300 rounds, reaching a given accuracy level in fewer communication rounds. FedAdam gradually closes the gap and the two methods reach statistically indistinguishable steady-state accuracy by round 500 (Table 2), while FedDyn remains substantially behind across the entire trajectory, most visibly in the validation loss curve, which stays above 1.5 even at round 500.

**Fig 4:**
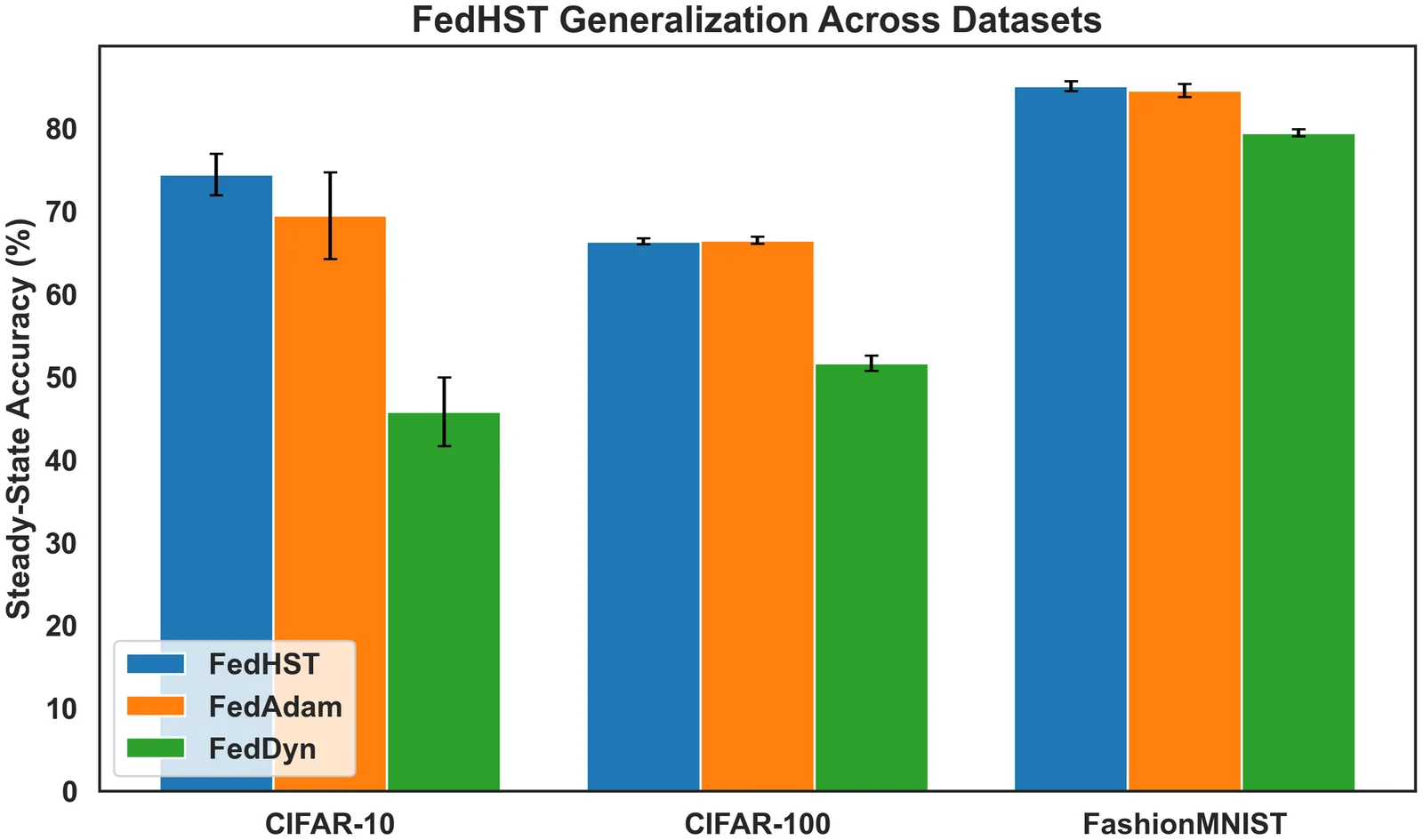
FedHST generalization across datasets. Steady-state accuracy (mean ±} std over *n*=3 seeds) for FedHST, FedAdam, and FedDyn on CIFAR-10, CIFAR-100, and FashionMNIST, summarizing the per-dataset trajectories of Figs 1 and 2 into a single comparison. FedHST’s largest margin over FedAdam appears on CIFAR-10, where it also carries a visibly smaller error bar, indicating both a higher mean accuracy and more consistent behavior across seeds. On CIFAR-100 the two methods are within a fraction of a percentage point of each other, consistent with the statistical parity reported in Table 2, while on FashionMNIST FedHST holds a small but consistent lead. FedDyn trails both adaptive methods on every dataset, with its gap widest on CIFAR-10 and narrowest on FashionMNIST, mirroring the convergence behavior discussed for Fig 1.

**Fig 5:**
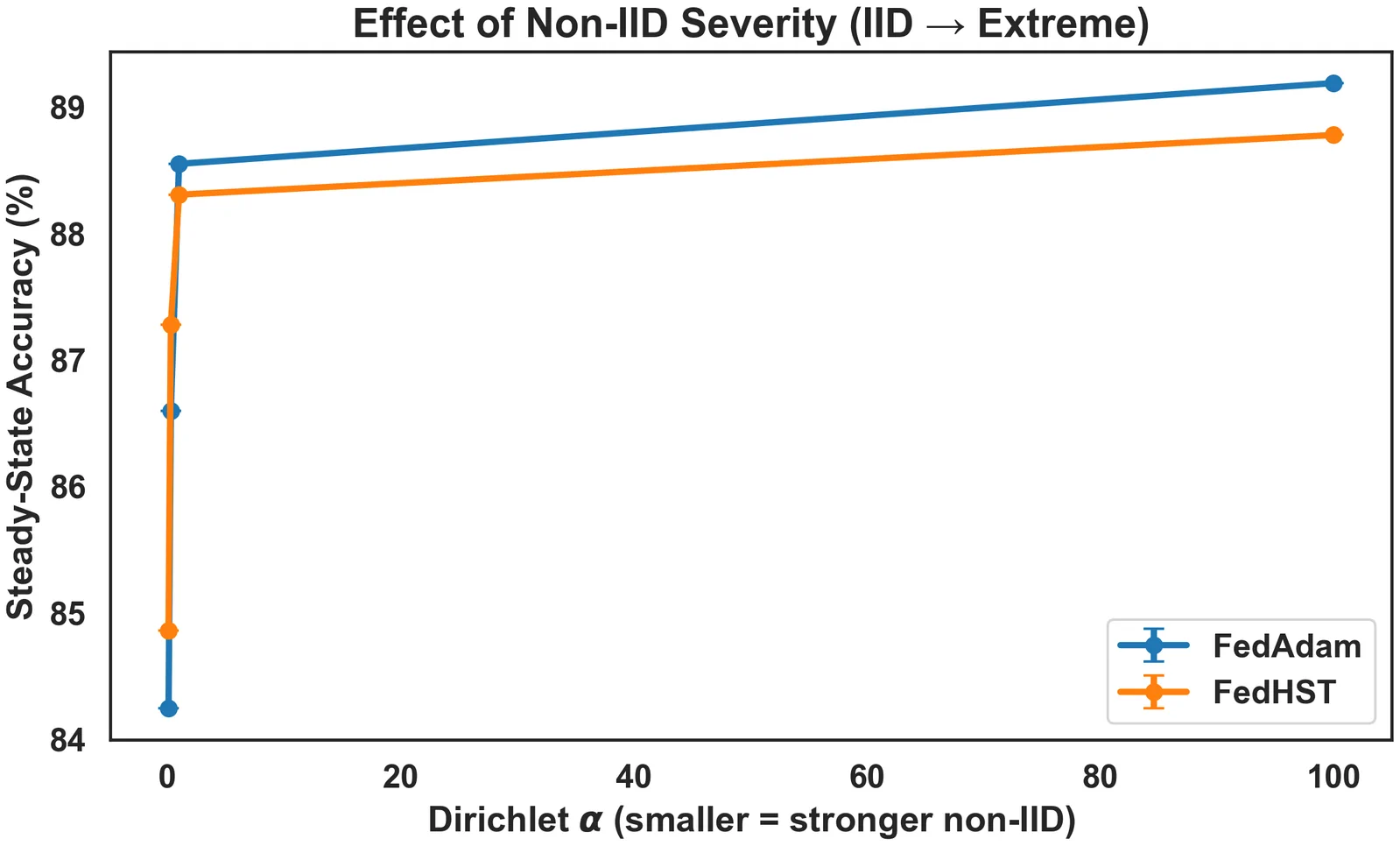
Effect of non-IID severity on FedHST vs. FedAdam. Steady-state accuracy on FashionMNIST as the Dirichlet concentration parameter α is relaxed from extreme heterogeneity (α=0.1) toward near-IID (α=100). Both methods improve sharply as α increases from 0.1 to around 1, after which gains flatten, reflecting that most of the difficulty in this setting comes from the extreme label skew rather than from moderate heterogeneity. FedHST’s advantage over FedAdam is largest precisely in the most heterogeneous regime (α≤1) and narrows as the partition approaches IID, consistent with the framework’s motivation: the spatial-variance damping term is designed to counteract client-update conflict that is most severe under strong non-IID partitioning and becomes progressively less relevant as client distributions converge.

Taken together, these trajectories show that FedHST’s advantage is most pronounced during the early-to-mid stages of training on the harder, more heterogeneous benchmarks (CIFAR-10 and CIFAR-100), while all three methods converge to comparable macro F1-scores on FashionMNIST, where the classification task itself is less sensitive to aggregation strategy. FedDyn’s wider variance bands across all three datasets are consistent with its known sensitivity to the regularization strength $\alpha_{\text{FedDyn}}$, discussed further in the Limitations subsection.
