# Supplementary material for: FedHST: Federated Hybrid Spatial–Temporal Optimization for robust non-IID learning
Source: PLoS One. 2026 Nov 12;21(11):e0360546. doi: 10.1371/journal.pone.0360546 (PMC13759210; doi:10.1371/journal.pone.0360546)
Supplement: Supplementary file 1 [file pone.0360546.s001.pdf]

PLOS One

FedHST: Federated Hybrid Spatial–Temporal Optimization for Robust Non-IID Learning

--Manuscript Draft--

**Manuscript Number:**	PONE-D-26-28169
**Article Type:**	Research Article
**Full Title:**	FedHST: Federated Hybrid Spatial–Temporal Optimization for Robust Non-IID Learning
**Short Title:**	FedHST: Federated Hybrid Spatial–Temporal Optimization for Robust Non-IID Learning
**Corresponding Author:**	Duy-Dong Le, MsC<br>University of Economics Ho Chi Minh City<br>Vinh long, Vinh Long VIET NAM
**Keywords:**	
**Abstract:**	Server-side optimization paradigms in Federated Learning (FL), such as FedAvgM and FedAdam, accelerate convergence but remain ``spatially blind'' to instantaneous cross-client directional conflicts under statistical heterogeneity (non-IID data). This limitation causes step-size explosions and severely destabilizes global aggregation. To address this, we propose \textbf{FedHST} (Federated Hybrid Spatial–Temporal Optimization), a novel framework featuring a dual-regulation engine. By integrating cross-client spatial variance as an adaptive damper alongside temporal tracking, FedHST dynamically compresses updates along high-friction coordinates while accelerating consensus-driven dimensions. Theoretically, we prove a non-convex convergence rate of $\mathcal{O}(1/\sqrt{T})$. Empirically, extensive evaluations on 100 clients under extreme non-IID constraints ($\alpha \in \{0.1, 0.3\}$) demonstrate that FedHST consistently outperforms FedAdam and FedAvgM across MNIST, FashionMNIST, and CIFAR-10 datasets. Notably, FedHST significantly reduces global validation standard deviation while achieving superior steady-state accuracy and f1-score, establishing a robust paradigm for adaptive federated optimization.
**Order of Authors:**	Duy-Dong Le, MsC
	Thai-Thinh Dang, MSc
**Additional Information:**	
**Question**	**Response**
**Financial Disclosure**<br><br>Enter a financial disclosure statement that describes the sources of funding for the work included in this submission. Review the submission guidelines for detailed requirements. View published research articles from *PLOS ONE* for specific examples.<br><br>This statement is required for submission and **will appear in the published article** if the submission is accepted. Please make sure it is accurate.	Yes

Powered by Editorial Manager® and ProduXion Manager® from Aries Systems Corporation

**Funded studies**<br>Enter a statement with the following details:<br>• Initials of the authors who received each award<br>• Grant numbers awarded to each author<br>• The full name of each funder<br>• URL of each funder website<br>• Did the sponsors or funders play any role in the study design, data collection and analysis, decision to publish, or preparation of the manuscript?<br><br>Did you receive funding for this work?	
Please add funding details.<br>as follow-up to "**Financial Disclosure**<br><br>Enter a financial disclosure statement that describes the sources of funding for the work included in this submission. Review the submission guidelines for detailed requirements. View published research articles from *PLOS ONE* for specific examples.<br><br>This statement is required for submission and **will appear in the published article** if the submission is accepted. Please make sure it is accurate.<br><br>**Funded studies**<br>Enter a statement with the following details:<br>• Initials of the authors who received each award<br>• Grant numbers awarded to each author<br>• The full name of each funder<br>• URL of each funder website<br>• Did the sponsors or funders play any role in the study design, data collection and analysis, decision to publish, or preparation of the manuscript?<br><br>Did you receive funding for this work?"	This research is funded and supported by the University of Economics Ho Chi Minh City (UEH), Vietnam.
Please select the country of your main research funder (please select carefully as in some cases this is used in fee calculation).	VIET NAM - VN

as follow-up to "**Financial Disclosure**<br><br>Enter a financial disclosure statement that describes the sources of funding for the work included in this submission. Review the submission guidelines for detailed requirements. View published research articles from *PLOS ONE* for specific examples.<br><br>This statement is required for submission and **will appear in the published article** if the submission is accepted. Please make sure it is accurate.<br><br>**Funded studies**<br>Enter a statement with the following details:<br>• Initials of the authors who received each award<br>• Grant numbers awarded to each author<br>• The full name of each funder<br>• URL of each funder website<br>• Did the sponsors or funders play any role in the study design, data collection and analysis, decision to publish, or preparation of the manuscript?<br><br>Did you receive funding for this work?"	
**Competing Interests**<br><br>Use the instructions below to enter a competing interest statement for this submission. On behalf of all authors, disclose any competing interests that could be perceived to bias this work—acknowledging all financial support and any other relevant financial or non-financial competing interests.<br><br>This statement is required for submission and **will appear in the published article** if the submission is accepted. Please make sure it is accurate and that any funding sources listed in your Funding Information later in the submission form are also declared in your Financial Disclosure statement.	The authors have declared that no competing interests exist.

View published research articles from *PLOS ONE* for specific examples.<br><br>**NO authors have competing interests**<br><br>Enter: *The authors have declared that no competing interests exist.*<br><br>**Authors with competing interests**<br><br>Enter competing interest details beginning with this statement:<br><br>*I have read the journal's policy and the authors of this manuscript have the following competing interests: [insert competing interests here]*<br><br>* typeset	
**Ethics Statement**<br><br>Enter an ethics statement for this submission. This statement is required if the study involved:<br><br>• Human participants<br>• Human specimens or tissue<br>• Vertebrate animals or cephalopods<br>• Vertebrate embryos or tissues<br>• Field research<br><br>Write "N/A" if the submission does not require an ethics statement.<br><br>General guidance is provided below. Consult the submission guidelines for detailed instructions. **Make sure that all information entered here is included in the Methods section of the manuscript.**	N/A We would like to clarify that our manuscript focuses strictly on theoretical Federated Learning optimization. The terms related to clinical or medical data in the text are purely academic examples to illustrate potential real-world applications of our framework. All empirical evaluations in this study were conducted exclusively using standard, publicly available, and fully anonymized benchmark datasets (MNIST, FashionMNIST, and CIFAR-10). No direct human participants or clinical trials were involved

**Format for specific study types**<br><br>**Human Subject Research (involving human participants and/or tissue)**<br>• Give the name of the institutional review board or ethics committee that approved the study<br>• Include the approval number and/or a statement indicating approval of this research<br>• Indicate the form of consent obtained (written/oral) or the reason that consent was not obtained (e.g. the data were analyzed anonymously)<br><br>**Animal Research (involving vertebrate animals, embryos or tissues)**<br>• Provide the name of the Institutional Animal Care and Use Committee (IACUC) or other relevant ethics board that reviewed the study protocol, and indicate whether they approved this research or granted a formal waiver of ethical approval<br>• Include an approval number if one was obtained<br>• If the study involved *non-human primates*, add *additional details* about animal welfare and steps taken to ameliorate suffering<br>• If anesthesia, euthanasia, or any kind of animal sacrifice is part of the study, include briefly which substances and/or methods were applied<br><br>**Field Research**<br><br>Include the following details if this study involves the collection of plant, animal, or other materials from a natural setting:<br>• Field permit number<br>• Name of the institution or relevant body that granted permission	
**Data Availability**<br><br>Before publication, Authors are required to make fully available and without restriction all data underlying their findings. Sharing the data underlying your findings increases the visibility, credibility, and impact of your work, while supporting transparency and future scientific discovery. Please see our PLOS Data	MNIST handwritten digit dataset is available at: http://yann.lecun.com/exdb/mnist/<br><br>FashionMNIST dataset is available at: https://github.com/zalandoresearch/fashion-mnist<br><br>CIFAR-10 dataset is available at: https://www.cs.toronto.edu/~kriz/cifar.html

Powered by Editorial Manager® and ProduXion Manager® from Aries Systems Corporation

Policy page for detailed information on this policy.<br><br>A **Data Availability Statement**, detailing where the data can be accessed, is required at first submission. Insert your Data Availability Statement in the box below.<br><br>Please see the data reporting section of our submission guidelines for instructions on what you need to include in your Data Availability Statement. Examples of acceptable Data Availability Statements are included here for reference:<br>1. The minimal data set [and accompanying code, where generated] is available at [repository name] via [DOI hyperlink]<br>2. No data was generated by this study. The following existing data sources were used: [dataset name] from [dataset location] available via [web address or DOI hyperlink].<br>3. For Study Protocols: No datasets were generated or analysed during the current study. All relevant data from this study will be made available upon study completion.<br><br>*PLOS allows rare exemptions to address legal and ethical concerns. If you have legal or ethical restrictions, please use the box below to detail these in full sentences for the Journal team to consider.*<br><br>This statement will be typeset if the manuscript is accepted for publication.	

Manuscript

Click here to access/download;Manuscript;FedHST_Plos_One.pdf

FedHST: Federated Hybrid Spatial–Temporal Optimization for Robust Non-IID Learning

Duy-Dong Le[1*], Thai-Thinh Dang[1],

1 University of Economics Ho Chi Minh City (UEH), Vietnam

dongld@ueh.edu.vn

Abstract

Server-side optimization paradigms in Federated Learning (FL), such as FedAvgM and FedAdam, accelerate convergence but remain "spatially blind" to instantaneous cross-client directional conflicts under statistical heterogeneity (non-IID data). This limitation causes step-size explosions and severely destabilizes global aggregation. To address this, we propose **FedHST** (Federated Hybrid Spatial–Temporal Optimization), a novel framework featuring a dual-regulation engine. By integrating cross-client spatial variance as an adaptive damper alongside temporal tracking, FedHST dynamically compresses updates along high-friction coordinates while accelerating consensus-driven dimensions. Theoretically, we prove a non-convex convergence rate of $\mathcal{O}(1/\sqrt{T})$. Empirically, extensive evaluations on 100 clients under extreme non-IID constraints ($\alpha \in \{0.1, 0.3\}$) demonstrate that FedHST consistently outperforms FedAdam and FedAvgM across MNIST, FashionMNIST, and CIFAR-10 datasets. Notably, FedHST significantly reduces global validation standard deviation while achieving superior steady-state accuracy and f1-score, establishing a robust paradigm for adaptive federated optimization.

Introduction

Context and the Paradigm Shift of Federated Learning

The explosive proliferation of Internet of Things (IoT) ecosystems, smart edge devices, wearable biomedical sensors, and distributed enterprise database architectures has catalyzed an unprecedented surge in the volume of data generated at the network edge. Extracting actionable knowledge and training high-capacity Deep Learning (DL) models from these distributed data streams is paramount for advancing artificial intelligence. However, conventional centralized training architectures necessitate the wholesale collection, transmission, and consolidation of raw edge data assets into centralized cloud storage infrastructures. This monolithic paradigm is increasingly unviable due to severe bandwidth constraints, high communication latency, and, most critically, mounting systemic risks concerning data confidentiality and user privacy [1].

Concurrently, global legal frameworks have evolved to penalize unauthorized data aggregation. Strict regulatory mandates, such as the European Union's General Data Protection Regulation (GDPR), the California Consumer Privacy Act (CCPA), and the Health Insurance Portability and

Accountability Act (HIPAA), impose heavy penalties on organizations that centralize sensitive user records. In response to these operational, ethical, and legal constraints, FL has emerged as a revolutionary decentralized machine learning paradigm [2]. FL radically restructures the model-training process by adhering to the core principle of moving the computational workload to the data rather than migrating the data to a centralized computational pool.

In a standard FL environment, a central orchestration server maintains a global parameter state while coordinating a massive network of edge clients. During each sequential communication round, the server broadcasts the current global model parameters to a selected subset of clients. Each participating client independently optimizes the model parameters on its local, private dataset using standard optimization techniques like Stochastic Gradient Descent (SGD) over multiple local epochs. Upon completing local computation, the clients transmit their updated model states or local parameter deltas (pseudo-gradients) back to the central server. The server then executes a server-side aggregation protocol—historically dominated by the Federated Averaging (FedAvg) algorithm—to fuse the local updates into an updated global model, which is subsequently broadcast to the clients for the next training iteration. This loop persists until the global architecture achieves convergence, ensuring that raw edge data never leaves its original local repository, thereby preserving privacy boundaries [3].

The Non-IID Challenge and the Mechanics of Client Drift

Despite its significant theoretical advantages for privacy preservation, the real-world deployment of FL encounters severe algorithmic bottlenecks. The most fundamental and mathematically challenging hurdle is the presence of statistical heterogeneity across decentralized client data repositories, commonly referred to as the non-IID (Not Identically and Independently Distributed) data challenge [4, 5]. In practical edge networks, the data generation habits of mobile phone users, the demographic profiles and clinical distributions within distinct regional medical centers, and the ambient environment of geographically scattered IoT sensors vary dramatically.

Mathematically, this implies that the local data distribution $\mathcal{D}_c$ at client c is highly distinct from the distribution $\mathcal{D}_j$ at client j, leading to severe variations across the network. These variations manifest across multiple distinct empirical dimensions [6]:

1. **Label Distribution Skew:** The marginal distributions of labels, $P_c(y)$, vary substantially across clients while the conditional distributions $P(x|y)$ remain uniform. This is exemplified by regional variations where certain classes are completely absent from local data stores.

2. **Feature Distribution Skew:** The marginal feature distributions $P_c(x)$ are highly heterogeneous across clients despite uniform label conditionals $P(y|x)$ (e.g., handwriting styles varying across distinct writers).

3. **Concept Drift:** The conditional distributions $P_c(y|x)$ vary across distinct clients even when feature marginals $P(x)$ are identical.

4. **Quantity Skew:** The total volume of local data samples $n_c = |\mathcal{D}_c|$

spans several orders of magnitude across the client fleet, causing highly unbalanced local gradients.

When statistical heterogeneity becomes severe—frequently modeled in algorithmic benchmarks using a symmetric Dirichlet distribution with an extremely restrictive concentration parameter ($\alpha \leq 0.1$) [7]—the local loss landscapes $F_c(w)$ of distinct clients diverge radically. Consequently, the local optimization trajectories are driven toward entirely separate, isolated local minima.

When clients execute multiple local optimization epochs ($E > 1$) to maximize communication efficiency and conserve network bandwidth, their respective weight vectors are pulled deep into their distinct local loss valleys. This structural misalignment is formally defined in the literature as the **Client Drift** phenomenon [8]. When the central server naively aggregates these severely drifted local parameter tensors via simple weighted averaging, the resulting global model is dragged into an sub-optimal intermediate state. This manifests empirically as severe convergence degradation, massive performance oscillations across successive rounds, and a catastrophic drop in the generalization accuracy of the global model.

The Spatial Blindness of Contemporary Server-Side Adaptive Optimizers

To mitigate the adverse effects of client drift without sacrificing communication efficiency, a prominent line of research has focused on transferring advanced stochastic optimization mechanics to the server side. This effort led to the formulation of server-side adaptive optimization frameworks, most notably the FedOpt family (comprising FedAdam, FedYogi, and FedAdagrad) [7]. These algorithms treat the average parameter delta aggregated from participating clients as a "pseudo-gradient" vector. By extending coordinate-wise adaptive learning rate algorithms (such as Adam or Yogi) to the server level, these methods dynamically regulate the global step-size for each parameter coordinate. They maintain historical moving averages of the first moment (momentum) and the temporal second moment (historical coordinate-wise variance) of the aggregated updates over successive communication rounds.

However, a deeper structural critique conducted in our study reveals a fundamental and highly problematic limitation within the mathematical architecture of all contemporary server-side adaptive federated optimizers: these formulations are entirely **spatially blind**. At any given global communication round t, the coordinate-wise step-size modifier of an optimizer like FedAdam depends exclusively on the historical temporal moving average v_t, which compiles the squares of the *mean* aggregated updates across the time domain $(t, t-1, t-2, \dots)$. Crucially, this temporal formulation contains absolutely no mathematical mechanism to detect or quantify the instantaneous cross-sectional variance or consensus across the client network within the current spatial layer.

This structural omission introduces severe vulnerability under extreme non-IID conditions. Consider a scenario where a specific model parameter coordinate is subject to intense conflict between two distinct factions of clients. Due to highly skewed local datasets, the first faction generates a large positive pseudo-gradient for this parameter, while the second faction generates an equally large negative update. When these updates are com-

bined at the server, their opposing forces cancel out, yielding a weighted mean update $\bar{\Delta}_t$ that approaches zero.

Because the historical second moment v_t relies solely on this mean update, it also shrinks toward zero over successive rounds. In the standard adaptive update rule, this second moment resides in the denominator. As $v_t \to 0$, the coordinate-wise step size is amplified by a factor of $1/(\sqrt{v_t} + \tau)$. Consequently, the server executes a massive, destabilizing update along that specific parameter coordinate. This occurs precisely when a prudent optimizer should minimize its step size due to the total absence of consensus across the client fleet. This spatial blindness triggers local gradient explosions, shatters the structural stability of the model's feature extraction layers, and induces severe performance drops.

Core Research Contributions

To systematically overcome the spatial blindness inherent in existing adaptive federated optimization methods, we present the design, theoretical proof, and empirical evaluation of the **FedHST (Federated Hybrid Spatial–Temporal Optimization)** framework. FedHST introduces a dual-regulation optimization engine that simultaneously accounts for temporal evolution and instantaneous spatial cross-client variance. The core scientific contributions of this work are summarized as follows:

1. **Formulation of the FedHST Optimization Engine:** We introduce a novel mathematical framework that incorporates both the temporal second moment of global updates and the instantaneous cross-client spatial variance into a unified coordinate-wise adaptive denominator. This creates a hybrid spatial-temporal scaling metric that automatically mitigates parameter-level client friction.

2. **Engineering of a Zero-Overhead Spatial Damper:** We prove that the instantaneous cross-client spatial variance tensor σ_t^{sp} can be computed completely at the server using the standard pseudo-gradients transmitted by clients. Consequently, FedHST enforces rigorous spatial damping without increasing client-side computational overhead, preserving network bandwidth and maintaining standard privacy guarantees.

3. **Rigorous Theoretical Convergence Proofs:** We present a comprehensive convergence analysis for FedHST under non-convex optimization assumptions, establishing an explicit convergence rate of $\mathcal{O}(1/\sqrt{T})$. Our proof structurally maps how the hybrid denominator stabilizes the optimization trajectory and bounds the residual variance under non-IID data regimes. This analysis builds upon recent advances in understanding data heterogeneity in federated optimization [9].

4. **Extensive Empirical Validation and Variance Reduction:** We evaluate FedHST through extensive empirical simulations on the MNIST, FashionMNIST, and CIFAR-10 benchmarks under extreme statistical heterogeneity ($\alpha = 0.1$ for MNIST/FashionMNIST; $\alpha = 0.3$ for CIFAR-10) distributed across a 100-client network. The results prove that FedHST achieves state-of-the-art accuracy and F1-scores

while reducing validation standard deviation by up to 61.6% compared to FedAdam and 86.8% compared to FedAvgM establishing exceptional numerical resilience.

The remainder of this article is organized as follows. Section systematically contextualizes our contribution within the existing federated optimization literature. Section formalizes the mathematical problem and the mechanics of federated aggregation. Section unpacks the algorithmic architecture and structural dynamics of FedHST. Section A provides full analytical proofs of convergence. Section details the experimental environment, while Section delivers comprehensive empirical analysis. Finally, Section outlines conclusions and strategic future work.

Related Work

The quest to achieve robust numerical convergence and suppress client drift within statistically heterogeneous FL environments has driven intensive research. The existing literature can be classified into three primary paradigms: Client-Side Regularization and Knowledge-Driven Distillation, Server-Side Adaptive Momentum Protocols, and Advanced Weighted Aggregation and Geometric Consensus Schemes.

Client-Side Regularization, Sparsity, and Knowledge Distillation

Client-side mitigation techniques focus on modifying the local optimization problem to prevent individual client updates from drifting too far from the global consensus topology.

The pioneering work in this space, FedProx [4], addresses statistical heterogeneity by introducing an isotropic ℓ_2 proximal regularization term into the local objective function:

$$\min_{w} h_c(w) = F_c(w) + \frac{\mu}{2} \|w - w_t\|^2 \tag{1}$$

This proximal term penalizes large deviations from the global state w_t broadcast at the beginning of the round. While mathematically straightforward, FedProx exhibits notable practical limitations. The isotropic regularization coefficient μ is highly sensitive and requires meticulous tuning; setting μ too small fails to counteract client drift, whereas setting it too large restricts local representation learning capability, locking parameters into suboptimal global states.

To bypass heuristic penalties, SCAFFOLD [8] leverages stateful control variates to approximate and counteract the client-specific drift vector during local training loops. Similarly, FedDyn [10] implements a dynamic regularization scheme based on sequential over-relaxation and Lagrangian multipliers to align local stationary points with the global optimum. However, these architectures introduce significant dual-tensor communication bottlenecks or persistent local memory overhead, making them unviable for bandwidth-constrained, resource-limited edge nodes.

To eliminate these overheads while preservation-learning under extreme data skew, recent paradigms have shifted toward structural sparsity combined with localized knowledge synthesis. Notably, SelfDistillCore [11] introduces an advanced federated framework that integrates top-k sparse

delta updates with historical knowledge integration via self-distillation. By transferring only compressed parameter vectors and deploying internal distillation blocks to maintain historical structural consistency, SelfDistillCore successfully circumvents the heavy transmission bottlenecks of control-variate methods while sustaining high tracking accuracy across non-IID client domains.

Server-Side Adaptive Engines and Momentum-Based Protocols

In contrast to client-side interventions, server-side adaptive aggregation protocols leave the local optimization loops unaltered, offloading the computational complexity of mitigating heterogeneity entirely to the central server. This paradigm initiated with Federated Averaging with Momentum (FedAvgM) [12], which applies heavy-ball temporal momentum to the aggregated pseudo-gradient. Mathematically, the server momentum buffer acts as a temporal low-pass filter, smoothing out the high-frequency gradient oscillations induced by conflicting local loss landscapes. Subsequent variants have integrated Nesterov accelerated gradient variants and decoupled client-server tracking frameworks (e.g., Mime [13]) to ensure that the global optimization trajectory can safely navigate through noisy local valleys.

The server-side approach was substantially advanced by the formalization of the FedOpt adaptive framework [7]. This framework introduced coordinate-wise adaptive learning rates into the federated aggregation engine, establishing algorithms such as FedAdagrad, FedYogi, and FedAdam. These methods utilize the historically accumulated temporal second moment of the aggregated mean pseudo-gradients to dynamically downscale updates along coordinate directions characterized by high temporal volatility. Recent studies have continuously extended this adaptive boundary; for instance, FedAdamom [14] introduces a dynamic momentum hyperparameter tuning mechanism to stabilize late-stage asymptotic convergence and maximize generalization performance under skewed data distributions.

Nevertheless, the entire lineage of server momentum and FedOpt adaptive architectures remains fundamentally constrained by its reliance on a purely temporal historical lens. Because these algorithms evaluate variance solely through the time series of the mean aggregated updates across successive rounds, they exhibit complete spatial blindness—lacking the intra-round awareness required to detect high cross-client variance within a single communication loop. Consequently, under severe non-IID conditions where local clients generate highly polarized update vectors that accidentally cancel each other out in the mean aggregator, this spatial blindness leads to severe step-size explosions, gradient misalignment, and catastrophic training instability.

Advanced Weighted Aggregation and Geometric Consensus Schemes

To address the limitations of naive average aggregation, an emerging domain focuses on redefining how client contributions are weighted and filtered at the server level. Standard federated systems rely exclusively on sample-size proportions (p_c), which frequently misrepresents the true optimization value of highly specialized local datasets under extreme label imbalance. To resolve this structural flaw, FedNolowe [15] introduces a normalized

loss-based weighted aggregation strategy. By dynamically transforming client aggregation weights as a function of their normalized local empirical losses, FedNolowe establishes a robust, sample-agnostic defense layer that inherently dampens the influence of noisy, divergent clients while amplifying structurally sound updates without requiring heavy client-side regularization overhead.

Concurrently, geometric consensus-driven methods have emerged to identify and isolate anomalous updates using multi-angle metrics. For instance, FedSCS [16] embeds stable cosine similarity scoring into the server loop to selectively filter out clients presenting highly divergent trajectories relative to the global historical path. Similarly, PNCS [17] fuses power-norm scales with multi-angle cosine vectors to maximize statistical diversity, while layer-wise agnostic schemes [18] dissect consensus metrics independently across distinct structural layers of the neural network topology to reject catastrophic non-IID noise.

However, these geometric consensus and rejection-based frameworks are engineered primarily as discrete, binary client selection filters or outlier rejection mechanisms. They do not provide a continuous, coordinate-wise step-size modifier. Consequently, they risk permanently discarding highly valuable, unique private features from specialized edge nodes simply because those nodes reside in distinct parameter sub-domains.

FedHST departs fundamentally from both discrete geometric filters and global loss-weighting schemes. Instead of uniformly down-weighting or discarding highly variant client assets, FedHST fully retains all client data updates. It utilizes the coordinate-wise cross-client spatial variance as a continuous, micro-level regulator embedded directly within the adaptive denominator of the server optimizer. This hybrid coupling provides an elegant, robust mathematical mechanism that synthesizes temporal history with intra-round spatial diversity to achieve unprecedented stability under extreme statistical heterogeneity.

Mathematical Preliminaries and Problem Formulation

The Federated Optimization Landscape

We formalize a standard FL environment consisting of a single central server orchestration node and a finite fleet of K decentralized edge clients. Each individual client $c \in \{1, 2, \ldots, K\}$ possesses an independent, private local dataset denoted as $\mathcal{D}_c$, with its cardinality given by $n_c = |\mathcal{D}_c|$. The total number of data samples distributed across the entire network is denoted by $N = \sum_{c=1}^{K} n_c$. We define the relative statistical weight of each client as $p_c = n_c/N$, satisfying the convex allocation condition $\sum_{c=1}^{K} p_c = 1$. The fundamental objective of the global system is to solve the empirical risk minimization problem over a continuous parameter space $w \in \mathbb{R}^d$:

$$\min_{w \in \mathbb{R}^d} f(w) = \sum_{c=1}^{K} p_c F_c(w) \tag{2}$$

The local objective function $F_c(w)$ characterizes the empirical loss of the model parameter state w evaluated over the local private data distribution

$\mathcal{D}_c$, and is defined via:

$$F_c(w) = \frac{1}{n_c} \sum_{x \in \mathcal{D}_c} \ell(w; x) \tag{3}$$

where $\ell(w; x)$ represents a smooth, non-convex loss operator (e.g., categorical cross-entropy) mapping the prediction error of the model parameterized by w on a data sample vector x. Under a non-IID data distribution, the local data distributions are statistically distinct, meaning $\mathbb{E}_{x\sim\mathcal{D}_c}[x] \neq \mathbb{E}_{x\sim\mathcal{D}_j}[x]$ for $c \neq j$. Consequently, the local objectives diverge, and their respective gradient fields are highly mismatched:

$$\nabla F_c(w) \neq \nabla F_j(w), \quad \forall w \in \mathbb{R}^d, \quad c \neq j \tag{4}$$

The Execution and Communication Loop

The optimization process proceeds through sequential global communication rounds indexed by $t = 1, 2, \ldots, T$. At the start of round t, the central server selects an active subset of clients, denoted by $S_t \subseteq \{1, 2, \ldots, K\}$, with a fixed sample cardinality $|S_t| = n_t$. The server then broadcasts the current global model parameter state $w_t \in \mathbb{R}^d$ to all selected nodes.

Upon receiving w_t, each selected client $c \in S_t$ initializes its local model parameters as $w^c_{t,0} = w_t$. The client then executes E local epochs of Stochastic Gradient Descent (SGD). Let $\tau \in \{0, 1, \ldots, E-1\}$ denote the local iteration index. The update step for the local model parameters is governed by:

$$w^c_{t,\tau+1} = w^c_{t,\tau} - \eta_l g^c_{t,\tau} \tag{5}$$

where $\eta_l > 0$ represents the local client learning rate, and $g^c_{t,\tau} = \nabla F_c(w^c_{t,\tau}; \xi^c_{t,\tau})$ denotes the stochastic gradient computed on a mini-batch $\xi^c_{t,\tau}$ sampled randomly from the local repository $\mathcal{D}_c$. After completing E local training epochs, the client computes its total update vector, defined as the **Pseudo-Gradient Δ^c_t**:

$$\Delta^c_t = w^c_{t,E} - w_t = -\eta_l \sum_{\tau=0}^{E-1} \nabla F_c(w^c_{t,\tau}; \xi^c_{t,\tau}) \tag{6}$$

Each participating client $c \in S_t$ transmits its pseudo-gradient Δ^c_t back to the central server over the communication channel. The server then computes the weighted mean pseudo-gradient update:

$$\bar{\Delta}_t = \frac{\sum_{c \in S_t} p_c \Delta^c_t}{\sum_{c \in S_t} p_c} \tag{7}$$

In standard server-side adaptive optimizers like FedAdam, this mean update $\bar{\Delta}_t$ is used to track historical moving averages across the time domain. As established previously, this process ignores the cross-sectional variation among individual client updates within the current communication round.

The Proposed FedHST Framework

Mathematical Definition of Spatial Variance

To overcome the limitations of spatial blindness, FedHST introduces a dedicated tracking operator at the server level to compute the instantaneous

cross-sectional consensus across the client fleet. We define the coordinate-wise **Spatial Variance** tensor, denoted by $\sigma_t^{\text{sp}} \in \mathbb{R}^d$, as follows:

$$\sigma_t^{\text{sp}} = \frac{\sum_{c \in S_t} p_c \left(\Delta_t^c - \bar{\Delta}_t\right)^{\odot 2}}{\sum_{c \in S_t} p_c} \tag{8}$$

where $\odot 2$ denotes the element-wise (Hadamard) squaring operator. For each individual parameter coordinate $j \in \{1, 2, \ldots, d\}$, the scalar component is evaluated via:

$$\sigma_t^{\text{sp}}[j] = \frac{\sum_{c \in S_t} p_c \left(\Delta_t^c[j] - \bar{\Delta}_t[j]\right)^2}{\sum_{c \in S_t} p_c} \tag{9}$$

The spatial variance tensor provides a direct, coordinate-wise metric of client disagreement within the current communication round. If all participating clients generate identical updates for a specific parameter coordinate j, then $\sigma_t^{\text{sp}}[j] = 0$, indicating a perfect network consensus. Conversely, if the updates $\Delta_t^c[j]$ diverge widely due to severe local data heterogeneity, $\sigma_t^{\text{sp}}[j]$ increases significantly, signaling severe coordinate-wise client drift and directional conflict.

The Hybrid Spatial-Temporal Coupling Operator

FedHST combines this instantaneous spatial variance tensor with the historically accumulated temporal tracking parameters. The central server maintains two distinct temporal moving average buffers:

$$m_t = \beta_1 m_{t-1} + (1 - \beta_1)\bar{\Delta}_t \tag{10}$$

$$v_t = \beta_2 v_{t-1} + (1 - \beta_2)\bar{\Delta}_t^{\odot 2} \tag{11}$$

where $\beta_1, \beta_2 \in [0, 1)$ represent the exponential decay constants for the first (momentum) and temporal second moments, respectively.

We construct the **Hybrid Spatial-Temporal Variance** operator, denoted by $V_{\text{hybrid}} \in \mathbb{R}^d$, by coupling these components through a linear combination:

$$V_{\text{hybrid}} = v_t + \gamma \sigma_t^{\text{sp}} \tag{12}$$

where $\gamma \geq 0$ is a scalar hyperparameter termed the **Spatial Damper Weight**. This parameter controls the sensitivity of the global optimization loop to cross-client statistical conflicts. The complete global model parameter state transition rule for round $t + 1$ is then defined as:

$$w_{t+1} = w_t + \eta \cdot \frac{m_t}{\sqrt{v_t + \gamma \sigma_t^{\text{sp}}} + \tau} \tag{13}$$

where $\eta > 0$ represents the adaptive global learning rate, $\tau > 0$ is an infinitesimal smoothing parameter ensuring numerical stability, and all fraction and square-root operations are executed element-wise.

Self-Regulation and Damping Mechanics

The update rule of FedHST introduces an elegant self-regulating mechanism for distributed optimization under non-IID regimes. To see this clearly, let us analyze the scalar update step for an arbitrary coordinate j at round t:

$$\text{Step}_t[j] = \frac{\eta \cdot m_t[j]}{\sqrt{v_t[j] + \gamma \sigma_t^{\text{sp}}[j]} + \tau} \tag{14}$$

We consider two distinct edge cases that characterize the behavior of this hybrid formulation:

- **Case 1: Severe Cross-Client Spatial Conflict ($\sigma_t^{\mathbf{sp}}[j] \gg 0$, $\bar{\Delta}_t[j] \to 0$):** This scenario typically arises in highly skewed non-IID settings. When different client clusters generate opposing updates of equal magnitude, their weighted mean $\bar{\Delta}_t[j]$ cancels out and vanishes toward zero. Under standard FedAdam optimization ($\gamma = 0$), this causes the temporal second moment $v_t[j]$ to shrink rapidly over successive rounds. As $v_t[j] \to 0$, the adaptive step size increases significantly due to the division by a vanishing denominator. This induces step-size explosions along highly volatile dimensions, leading to training instability and client drift.

  In contrast, FedHST identifies this spatial conflict immediately. The large variance among individual client updates causes $\sigma_t^{\text{sp}}[j]$ to increase sharply. When incorporated into the denominator via $\gamma\sigma_t^{\text{sp}}[j]$, it counteracts the shrinking of $v_t[j]$, acting as an **automatic numerical brake**. This effectively dampens the step size along highly disputed dimensions, protecting the structural integrity of the model layers and stabilizing the global trajectory.

- **Case 2: High Network Consensus ($\sigma_t^{\mathbf{sp}}[j] \to 0$):** This scenario occurs when the global model enters a well-aligned optimization valley or operates in a statistically homogeneous (IID) data environment. Here, the individual client updates align closely with the global consensus, meaning $\Delta_t^c[j] \approx \bar{\Delta}_t[j]$ for all participating nodes. Consequently, the cross-client spatial variance $\sigma_t^{\text{sp}}[j]$ vanishes toward zero.

  As $\sigma_t^{\text{sp}}[j] \to 0$, the spatial damping term automatically deactivates, and the hybrid operator converges to the standard temporal second moment ($V_{\text{hybrid}} \to v_t$). This seamlessly transitions FedHST back into a standard temporal adaptive optimizer like FedAdam, allowing it to safely accelerate optimization along coordinates characterized by high consensus and speed up overall convergence.

The complete procedural architecture of FedHST is formalized below in Algorithm 1.

Experimental Setup

We validate the performance of the FedHST framework through extensive empirical simulations under controlled non-IID data regimes.

Dataset Profiles and Statistical Partitioning

Our evaluation utilizes three prominent vision benchmarks with distinct loss surface characteristics:

1. **MNIST:** A standard dataset of handwritten digits containing 70,000 28×28 grayscale images across 10 balanced classes (0 through 9). MNIST possesses well-defined cluster boundaries and a relatively smooth loss landscape.

Algorithm 1 FedHST: Federated Hybrid Spatial–Temporal Optimization Engine

Require: Rounds T, local epochs E, learning rates η_l, η, decay parameters β_1, β_2, spatial weight γ, stabilizer τ.

Server Execution:
Initialize global model $w_1 \in \mathbb{R}^d$, buffers $m_0 \leftarrow \mathbf{0}$, $v_0 \leftarrow \mathbf{0}$.
for $t = 1$ to T **do**
  Server samples a random subset of clients $S_t \subseteq \{1, \ldots, K\}$.
  for each client $c \in S_t$ **in parallel do**
    $\Delta_t^c \leftarrow$ **ClientLocalTraining**(c, w_t)
    Client c sends update Δ_t^c and data weight p_c to server.
  end for
  Compute aggregate mean: $\bar{\Delta}_t \leftarrow \sum_{c \in S_t} p_c \Delta_t^c \,/\, \sum_{c \in S_t} p_c$
  Compute spatial variance: $\sigma_t^{\mathrm{sp}} \leftarrow \sum_{c \in S_t} p_c \left(\Delta_t^c - \bar{\Delta}_t\right)^{\odot 2} \,/\, \sum_{c \in S_t} p_c$
  Update temporal buffers:
    $m_t \leftarrow \beta_1 m_{t-1} + (1 - \beta_1)\bar{\Delta}_t$
    $v_t \leftarrow \beta_2 v_{t-1} + (1 - \beta_2)\bar{\Delta}_t^{\odot 2}$
  Update global parameters: $w_{t+1} \leftarrow w_t + \eta \cdot \frac{m_t}{\sqrt{v_t + \gamma \sigma_t^{\mathrm{sp}}} + \tau}$
end for

Procedure ClientLocalTraining(c, w_t):
Initialize local model $w^c \leftarrow w_t$.
Perform E epochs of local SGD updates: $w^c \leftarrow w^c - \eta_l \nabla F_c(w^c; b)$ for mini-batches $b \in \mathcal{D}_c$.
return $\Delta_t^c \leftarrow w^c - w_t$

2. **FashionMNIST:** A more complex alternative featuring 70,000 28×28 grayscale images of clothing items across 10 classes (e.g., shirts, sneakers, coats). This dataset exhibits high intra-class variance and overlapping feature spaces, creating a challenging, highly non-convex loss surface with numerous local minima and saddle points.

3. **CIFAR-10:** A challenging RGB image dataset containing 60,000 32×32 color images across 10 classes, with higher intra-class variance and a more complex loss landscape than both MNIST and FashionMNIST.

To simulate severe statistical heterogeneity across the client fleet, we partition the training data using a symmetric Dirichlet distribution. For MNIST and FashionMNIST we use an extreme concentration parameter $\alpha = 0.1$ [7]. For CIFAR-10, we use $\alpha = 0.3$ to maintain a sufficient number of samples per client while still inducing strong label skew. For each individual client $c \in \{1, \ldots, 100\}$, a class probability vector q_c is sampled from the Dirichlet distribution:

$$q_c \sim \mathrm{Dirichlet}(\alpha \cdot \mathbf{1}_{10}) \tag{15}$$

Data samples are then assigned to clients without replacement according to these probabilities. Setting $\alpha = 0.1$ induces extreme label distribution skew across the network. Under this configuration, typical clients receive samples from only 2 or 3 distinct classes, while the remaining classes are entirely unrepresented in their local repositories. This sharp partition creates intense directional conflict between client updates during global

aggregation, challenging the stability of the optimizers. The Dirichlet-based partition methodology is widely adopted in the FL literature for its ability to control label skew systematically [19].

Neural Network Architectures

For MNIST and FashionMNIST we utilize a standard Convolutional Neural Network (`SimpleCNN`) architecture with two convolutional layers followed by max-pooling and a fully connected output layer, as described in the original FedAvg paper [2]. This ensures that performance differences stem entirely from the choice of server-side aggregation protocol rather than architectural heuristics.

For CIFAR-10 we employ a more powerful `CIFAR10CNN` architecture designed to handle the complexity of RGB images:

- Conv2d(3,64,3, padding=1) + GroupNorm(8,64) + ReLU
- Conv2d(64,64,3, padding=1) + GroupNorm(8,64) + ReLU + MaxPool2d(2)
- Conv2d(64,128,3, padding=1) + GroupNorm(16,128) + ReLU + MaxPool2d(2)
- Flatten + Linear(128*8*8, 1024) + ReLU + Dropout(0.4) + Linear(1024,10)

This architecture provides sufficient capacity for CIFAR-10 classification while maintaining computational efficiency.

Algorithmic Baselines and Hyperparameter Synchronization

The system is evaluated over a total duration of $T = 500$ global communication rounds for CIFAR-10 (and 50 rounds for MNIST/FashionMNIST in the original study). In each round, the server samples a random fraction of the network, selecting $n_t = 20$–30 clients to participate. Participating nodes execute $E = 5$ local training epochs on their local data stores using standard mini-batch SGD with a batch size of 64 and a fixed local learning rate $\eta_l = 0.01$.

To isolate the impact of spatial damping, all server-side hyperparameters are synchronized across the baseline algorithms:

- **FedAvgM (Federated Averaging with Momentum) [12]:** Server learning rate $\eta = 1.0$; heavy-ball momentum coefficient $\beta = 0.9$.
- **FedAdam (Adaptive Federated Optimization) [7]:** Server learning rate $\eta = 0.01$; momentum decay $\beta_1 = 0.9$; temporal variance decay $\beta_2 = 0.999$; numerical stabilization constant $\tau = 10^{-3}$.
- **FedHST (Proposed):** Server learning rate $\eta = 0.01$; momentum decay $\beta_1 = 0.9$; temporal variance decay $\beta_2 = 0.999$; numerical stabilization constant $\tau = 10^{-3}$; Spatial Damper Weight $\gamma = 0.5$.

By setting $\gamma = 0.5$, FedHST balances temporal momentum with instantaneous cross-client spatial variance. When $\gamma = 0$, the algorithm simplifies exactly to standard FedAdam.

All experimental scenarios are executed across two independent random seeds (84, 42) to verify reproducibility. Performance metrics are evaluated comprehensively across the full global validation dataset after each communication round. To measure performance under steady-state conditions, we compute the empirical mean and standard deviation (±std) across the final 15 communication rounds (rounds 35 to 50) for MNIST and FashionMNIST, and across the final 50 rounds (rounds 451 to 500) for CIFAR-10. This evaluation window provides a robust assessment of long-term numerical stability and convergence quality under statistical heterogeneity.

Results and Empirical Analysis

In this section, we conduct a rigorous empirical evaluation of the proposed FedHST framework against two state-of-the-art baselines: FedAvgM and FedAdam. To assess the algorithmic resilience under severe non-IID conditions, we systematically advance our analysis from a smooth digit recognition landscape to highly complex object classification environments.

Quantitative Performance on MNIST

We first evaluate the algorithms on the MNIST dataset to analyze their fundamental behavior on a relatively smooth, well-behaved loss landscape. The quantitative metrics accumulated over the final steady-state window are summarized in Table 1, and the corresponding convergence trajectories are illustrated in Fig 1.

Table 1: **Performance Comparison on the MNIST Dataset ($\alpha = 0.1$) Over the Final Steady-State Window (Rounds 35–50).**

**Algorithm**	**Validation Accuracy (%)**	**Macro F1-Score**	**Validation Cross-Entropy Loss**
FedAvgM	95.75 ± 1.60	0.9569 ± 0.0169	0.1369 ± 0.0569
FedAdam	96.31 ± 0.69	0.9630 ± 0.0071	**0.1139 ± 0.0215**
**FedHST (Ours)**	**96.41 ± 0.76**	**0.9639 ± 0.0077**	0.1167 ± 0.0208

Table notes: Values report the Mean ± Standard Deviation calculated across the final 15 communication rounds. Bold text highlights the top-performing framework for each evaluation metric.

On the simpler MNIST dataset, FedHST and FedAdam display comparable optimization performance, with validation accuracies reaching 96.41% and 96.31%, respectively. Both adaptive methods consistently outperform the traditional FedAvgM baseline (95.75%). The validation loss profiles for FedHST (0.1167) and FedAdam (0.1139) also trace remarkably parallel paths.

Fig 1. Convergence trajectories on the MNIST dataset ($\alpha = 0.1$). The learning curves track the evolution of the Macro F1-Score and Validation Cross-Entropy Loss across 50 communication rounds, validating the baseline optimization flow under mild structural variations.

This behavior explicitly confirms the adaptive properties of the FedHST framework. Because the MNIST handwritten digits possess highly uniform geometric structures and sparse pixel activations, the individual client

update vectors align closely even under extreme label skew ($\alpha = 0.1$). This causes the cross-client spatial variance tensor to naturally diminish toward zero ($\sigma_t^{\text{sp}} \to 0$). As a result, the spatial damping term gracefully deactivates, allowing FedHST to function identically to standard FedAdam and maintain rapid convergence without introducing unnecessary regularization overhead.

Quantitative Performance on FashionMNIST

The FashionMNIST dataset, characterized by its highly intricate non-convex loss surface and diverse morphological clothing features, provides a much more challenging environment to evaluate the tracking stability of federated optimizers. The performance metrics accumulated over the steady-state window are summarized in Table 2.

Table 2: **Performance Comparison on the FashionMNIST Dataset ($\alpha = 0.1$) Over the Final Steady-State Window (Rounds 35–50).**

**Algorithm**	**Validation Accuracy (%)**	**Macro F1-Score**	**Validation Cross-Entropy Loss**
FedAvgM	77.02 ± 6.58	0.7577 ± 0.0743	0.9253 ± 0.4963
FedAdam	82.07 ± 2.27	0.8127 ± 0.0253	0.5241 ± 0.0739
**FedHST (Ours)**	**84.76 ± 0.87**	**0.8430 ± 0.0100**	**0.4302 ± 0.0250**

Table notes: Values report the Mean ± Standard Deviation calculated across the final 15 communication rounds. Bold text highlights the top-performing framework for each evaluation metric.

The empirical numbers demonstrate the clear superiority of our proposed framework. As visualized in the convergence trajectories in Fig 2, FedHST achieves a peak mean validation accuracy of 84.76%, outperforming standard FedAdam by 2.69 percentage points and substantially improving upon the classic FedAvgM approach by 7.74 percentage points. This gain is strongly mirrored in the Macro F1-score, where FedHST reaches 0.8430, showing highly robust representation learning across statistically heterogeneous client distributions.

Fig 2. Convergence trajectories on the FashionMNIST dataset ($\alpha = 0.1$). Curves depict the Macro F1-Score and Validation Cross-Entropy Loss over 50 rounds, highlighting FedHST's ability to suppress the intense volatility and loss spikes that cripple conventional server momentum architectures.

Crucially, the standard deviation metrics highlight the exceptional numerical stability of our method. FedHST bounds its accuracy variance to a remarkably tight ±0.87%. In comparison, standard FedAdam exhibits an accuracy variance of ±2.27%, while FedAvgM shows severe systemic instability with a variance of ±6.58%. These results confirm that FedHST reduces performance volatility by over 61.6% compared to FedAdam. This stabilization is further verified by the validation cross-entropy loss profile, where FedHST maintains a smooth, well-bounded trajectory (0.4302 ± 0.0250), successfully filtering out the destructive client drift that causes severe gradient oscillations in alternative frameworks.

Quantitative Performance on CIFAR-10

Moving beyond grayscale imagery, CIFAR-10 presents an even more arduous optimization landscape due to its multiple RGB channels, rich intra-class variance, and high background noise. Table 3 reports the performance statistics evaluated over the final extended window (rounds 451–500) under a strict Dirichlet allocation ($\alpha = 0.3$).

Table 3: **Performance Comparison on the CIFAR-10 Dataset ($\alpha = 0.3$) Over the Final Extended Window (Rounds 451–500).**

Algorithm	Validation Accuracy (%)	Macro F1-Score	Validation Cross-Entropy Loss
FedAvgM	63.08 ± 2.61	0.6250 ± 0.0281	1.0540 ± 0.0777
FedAdam	72.95 ± 1.20	0.7272 ± 0.0124	0.7751 ± 0.0306
**FedHST (Ours)**	**73.66 ± 0.98**	**0.7342 ± 0.0104**	**0.7528 ± 0.0247**

Table notes: Values report the Mean ± Standard Deviation calculated across the final 50 communication rounds of a 500-round long-term execution. Bold text highlights the top-performing framework for each evaluation metric.

As summarized in Table 3 and dynamically tracked over 500 communication rounds in Fig 3, FedHST achieves the highest mean accuracy of 73.66%, outperforming FedAdam by 0.71 percentage points and the non-adaptive FedAvgM baseline by a massive 10.58 percentage points. More importantly, the standard deviation of FedHST (0.98%) is significantly lower than that of FedAdam (1.20%) and FedAvgM (2.61%), representing an 18.3% variance reduction over FedAdam and a 62.5% reduction over FedAvgM.

Fig 3. Extended convergence trajectories on the complex CIFAR-10 dataset ($\alpha = 0.3$). The long-term metrics across 500 communication rounds demonstrate that FedHST consistently maintains steady asymptotic convergence and the lowest cross-entropy loss envelope without undergoing late-stage stagnation.

The macro F1-score follows an identical trend: FedHST achieves 0.7342, surpassing FedAdam (0.7272) and FedAvgM (0.6250). Furthermore, the validation cross-entropy loss of FedHST (0.7528 ± 0.0247) remains robustly minimized compared to FedAdam (0.7751 ± 0.0306) and FedAvgM (1.0540 ± 0.0777). These complex vision results conclusively validate the algorithmic efficiency of our hybrid historical state tracking mechanism, demonstrating that locking down high-conflict gradient dimensions preserves optimization speed while eliminating toxic momentum inertia in highly complex distributed learning systems.

Analysis of Convergence Trajectories

The round-by-round optimization paths across the 50 communication rounds for FashionMNIST and MNIST, and across 500 rounds for CIFAR-10, provide deeper insights into the learning dynamics of the frameworks.

On the complex FashionMNIST dataset, the training trajectory can be divided into three distinct phases:

Early Exploration Phase (Rounds 1 to 15): All three optimization methods exhibit a rapid initial increase in accuracy. However, FedAvgM begins to plateau early due to its lack of coordinate-wise learning rate

scaling, while the adaptive mechanisms of FedAdam and FedHST enable faster early learning.

Mid-Round Divergence Phase (Rounds 16 to 35): This phase reveals the impact of client drift as local models approach their distinct regional minima. The validation trajectory of FedAdam shows significant instability, characterized by sharp drops in accuracy and corresponding spikes in validation loss (e.g., between rounds 20 and 30). This instability highlights the spatial blindness of standard adaptive methods, which scale up step sizes along conflicting parameter dimensions when the mean update vanishes. In contrast, the trajectory of FedHST remains smooth and steady, as its spatial damping mechanism mitigates directional conflicts across the client fleet.

Steady-State Plateau Phase (Rounds 36 to 50): In the final phase, FedHST maintains a stable, high-performance plateau, whereas FedAdam continues to fluctuate and FedAvgM exhibits persistent oscillations.

On the CIFAR-10 dataset, the same pattern emerges over the extended 500-round horizon. FedHST consistently maintains a smooth ascending accuracy curve with minimal oscillations, whereas FedAdam exhibits periodic drops (e.g., around rounds 200–250) and FedAvgM shows high volatility throughout the entire training process. This behavior aligns with the theoretical analysis: the spatial variance term σ_t^{sp} automatically dampens step sizes when client updates conflict, preventing divergence and ensuring stable convergence even on complex RGB image classification tasks.

In-depth Discussion and Scientific Implications

Computational and Communication Complexity Analysis

A key advantage of FedHST is its algorithmic efficiency, achieving Pareto optimality across computation, communication, and privacy dimensions. We analyze these elements systematically below:

1. **Client-Side Complexity:** The local training loop remains entirely unmodified. Clients execute standard mini-batch SGD on their local data stores, incurring zero additional computational or memory overhead.

2. **Communication Payload:** The network bandwidth consumption matches standard federated optimization protocols exactly. Clients only transmit their local parameter updates $\Delta_t^c \in \mathbb{R}^d$. Unlike methods like SCAFFOLD [8], FedHST requires no transmission of high-dimensional control variates or secondary optimization state tensors, making it highly suitable for bandwidth-constrained edge channels.

3. **Server-Side Complexity:** The computation of the coordinate-wise spatial variance tensor $\sigma_t^{\text{sp}} = \frac{1}{n_t}\sum_{c \in S_t} p_c(\Delta_t^c - \bar{\Delta}_t)^{\odot 2}$ follows a linear complexity of $\mathcal{O}(n_t \cdot d)$, where n_t is the number of active clients and d is the parameter dimensionality. Because this operation consists of simple element-wise addition, subtraction, and multiplication, it can be efficiently parallelized on server GPU architectures, adding negligible runtime overhead.

Real-World Industrial Applications

The robustness and stability of FedHST under extreme statistical heterogeneity make it highly valuable for real-world distributed applications:

Decentralized Healthcare Networks: In medical imaging applications, separate diagnostic centers utilize distinct imaging hardware configurations and encounter unique regional patient demographics, creating severe non-IID conditions. Recent advances in FL for medical imaging have demonstrated the effectiveness of privacy-preserving FL frameworks in multi-modal medical image analysis [20]. The spatial damping mechanism of FedHST prevents global aggregation from destabilizing due to localized variations, ensuring a reliable and safe optimization path for critical medical diagnostic models.

Autonomous Driving and Intelligent Transportation Systems: Fleet vehicles operating across diverse urban environments collect highly heterogeneous environmental and situational data streams. FL has emerged as a key technology for enhancing vehicle intelligence, with frameworks such as iFLOW enabling dynamic multi-model asynchronous training while protecting data privacy [21]. FedHST provides a stable framework for aggregating these disparate edge insights at a central orchestration server, preventing performance drops and ensuring the robust development of vision and control models.

Conclusion

This work addresses a fundamental limitation in contemporary server-side adaptive federated optimizers: their spatial blindness to instantaneous cross-client conflicts within individual communication rounds. We introduced FedHST (Federated Hybrid Spatial–Temporal Optimization), a novel server-side aggregation engine that integrates historically accumulated temporal second moments with a coordinate-wise measure of cross-client spatial variance. This hybrid formulation acts as an automatic spatial damper, dynamically reducing global step sizes along highly contested parameter dimensions while accelerating optimization along directions of high consensus.

We validated the framework through non-convex convergence analysis, establishing an explicit convergence rate of $\mathcal{O}(1/\sqrt{T})$ and demonstrating how spatial damping actively moderates the optimization residual under statistical heterogeneity. Empirically, extensive evaluations were conducted under extreme non-IID configurations ($\alpha = 0.1$ for MNIST/FashionMNIST; $\alpha = 0.3$ for CIFAR-10) on the MNIST, FashionMNIST, and CIFAR-10 benchmarks. The results confirm that FedHST consistently outperforms state-of-the-art server-side optimizers, achieving a peak accuracy of 84.76% on FashionMNIST and 73.66% on CIFAR-10 while reducing validation performance variance by up to 61.6%. Crucially, these gains are achieved with zero additional overhead on client computation or communication bandwidth, offering a highly efficient and robust solution for heterogeneous FL environments.

Acknowledgments

This research is funded and supported by the University of Economics Ho Chi Minh City (UEH), Vietnam.

References

1. Chung W, Lo C, Lin Y, Chen Z, Hung C. Decentralized federated learning with non-IID data: Challenges, trends, and future opportunities. ACM Computing Surveys. 2025;58(8):1–41.

2. McMahan B, Moore E, Ramage D, Hampson S, Aguera y Arcas B. Communication-efficient learning of deep networks from decentralized data. In: Proceedings of the 20th International Conference on Artificial Intelligence and Statistics (AISTATS); 2017. p. 1273–1282.

3. Kairouz P, McMahan HB, Avent B, Aurier A, Baranjuk RG, Chen J, et al. Advances and open problems in federated learning. Foundations and Trends in Machine Learning. 2021;14(1–2):1–210.

4. Li T, Sahu AK, Zaheer M, Sanjabi M, Talwalkar A, Smith V. Federated optimization in heterogeneous networks. In: Proceedings of Machine Learning and Systems (MLSys); 2020. p. 429–450.

5. Tan AZ, Yu H, Cui L, Yang Q. Toward personalized federated learning. IEEE Transactions on Neural Networks and Learning Systems. 2022;34(12):9587–9603.

6. Federated graph neural networks in non-IID scenarios—A comprehensive survey. Neurocomputing. 2025;652:131007.

7. Reddi S, Charles Z, Zaheer M, Garrett Z, Rush K, Konečný J, et al. Adaptive federated optimization. In: International Conference on Learning Representations (ICLR); 2021.

8. Karimireddy SP, Kale S, Mohri M, Reddi S, Stich SU, Suresh AT. SCAFFOLD: Stochastic controlled averaging for federated learning. In: Proceedings of the 37th International Conference on Machine Learning (ICML); 2020. p. 5132–5143.

9. Wang J, Wang S, Chen RR, Ji M. A new theoretical perspective on data heterogeneity in federated optimization. In: Proceedings of the 40th International Conference on Machine Learning (ICML); 2023.

10. Acar DAE, Zhao Y, Navarro RM, Mattina M, Whatmough PN, Saligrama V. Federated learning based on dynamic regularization. In: International Conference on Learning Representations (ICLR); 2021.

11. Le DD. SelfDistillCore: a federated learning framework with sparse updates and historical knowledge integration. PeerJ Computer Science. 2026;.

12. Hsu TMH, Qi H, Brown M. Measuring the effects of non-identical data distribution for federated visual classification. arXiv preprint arXiv:190906335. 2019;.

13. Karimireddy SP, Jaggi M, Kale S, Mohri M, Reddi SJ, Stich SU, et al. Mime: Mimicking centralized first-order methods in federated learning. In: Proceedings of the International Conference on Machine Learning (ICML); 2020.

14. Hou W, Chen T, Wang F, Wu T, Zheng Z, Tang S, et al. FedAdamom: Adaptive momentum for improved generalization in federated optimization. In: Proceedings of the IEEE/CVF Conference on Computer Vision and Pattern Recognition (CVPR); 2026.

15. Le DD, Huynh TN, Tran AK, Dao MS, Bao PT. FedNolowe: A normalized loss-based weighted aggregation strategy for robust federated learning in heterogeneous environments. PLoS ONE. 2025;20(8):e0322766.

16. Haque RU, Markopoulos P. Robust Federated Learning via Stable Cosine Similarity. In: Proceedings of the 2025 IEEE International Carnahan Conference on Security Technology (ICCST); 2025. p. 1–6.

17. Li L, Liu Y, Ning Y, Sini S, Chen J. PNCS: Power-Norm Cosine Similarity for Diverse Client Selection in Federated Learning. In: Proceedings of the 2025 IEEE International Conference on Communications (ICC); 2025.

18. García-Márquez M, Rodríguez-Barroso N, Luzón MV, Herrera F. Improving (α,f)-Byzantine resilience in federated learning via layerwise aggregation and cosine distance. Knowledge-Based Systems. 2025;326:114004.

19. FedArtML: A tool to facilitate the generation of non-IID datasets in a controlled way to support federated learning research. IEEE Access. 2024;12:81004–81016.

20. FedVGM: Enhancing federated learning performance on multi-dataset medical images with XAI. IEEE Journal of Biomedical and Health Informatics. 2025;30(2):1272–1285.

21. Wang Q, Yao Y, Ammar N, Shi W. iFLOW: An intelligent and scalable multi-model federated learning framework on the wheels. IEEE Transactions on Intelligent Transportation Systems. 2025;.

A Rigorous Convergence Analysis

We establish theoretical convergence guarantees for FedHST under standard non-convex optimization assumptions. We first outline these foundational assumptions, derive essential supporting lemmas, and state the primary convergence theorem.

A.1 Foundational Mathematical Assumptions

To ensure mathematical tractability while reflecting realistic federated learning environments, we impose the following standard assumptions on the optimization landscape:

Assumption A.1 (Lipschitz Continuous Smoothness)**.** *The objective loss functions $F_c(w)$ of all individual decentralized clients $c \in \{1, \ldots, K\}$ are continuously differentiable and satisfy the Lipschitz smoothness condition with a uniform constant $L > 0$. That is, for any parameter pair $x, y \in \mathbb{R}^d$, we have:*

$$\|\nabla F_c(x) - \nabla F_c(y)\| \leq L \|x - y\| \tag{16}$$

By the linear properties of differentiation, the global aggregate loss function $f(w) = \sum_{c=1}^{K} p_c F_c(w)$ also satisfies the Lipschitz smoothness condition with the same uniform constant L:

$$\|\nabla f(x) - \nabla f(y)\| \leq L \|x - y\| \tag{17}$$

Assumption A.2 (Bounded Local Stochastic Variance)**.** *The stochastic gradient field computed on local mini-batches within any client node c exhibits an unbiased expectation relative to the true local gradient field, and its variance is bounded uniformly by a scalar constant $\sigma^2 \geq 0$:*

$$\mathbb{E}_{\xi \sim \mathcal{D}_c} \|\nabla F_c(w;\xi) - \nabla F_c(w)\|^2 \leq \sigma^2, \quad \forall w \in \mathbb{R}^d, \quad c \in \{1, \dots, K\} \tag{18}$$

Assumption A.3 (Bounded Statistical Heterogeneity)**.** *The cross-client variance of the true local gradient fields relative to the true global aggregate gradient field is bounded uniformly across the parameter space by a non-negative scalar constant $\sigma_H^2 \geq 0$:*

$$\sum_{c=1}^{K} p_c \|\nabla F_c(w) - \nabla f(w)\|^2 \leq \sigma_H^2, \quad \forall w \in \mathbb{R}^d \tag{19}$$

If the data distribution is perfectly homogeneous (IID), then $\sigma_H^2 = 0$. Larger values of σ_H^2 reflect increasing statistical heterogeneity across the client fleet.

Assumption A.4 (Unbiased Client Selection Scheme)**.** *At each communication round t, the central server samples an active client subset S_t uniformly at random from the total fleet. The selection process ensures that client inclusion probabilities match their relative data scale weights, satisfying the unbiased expectation condition:*

$$\mathbb{E}_{S_t}\left[\frac{\sum_{c \in S_t} p_c \chi_c}{\sum_{c \in S_t} p_c}\right] = \sum_{c=1}^{K} p_c \chi_c \tag{20}$$

for any arbitrary sequence of vectors $\{\chi_c\}_{c=1}^{K}$.

A.2 Essential Supporting Lemmas

Let $\mathbb{E}_t[\cdot]$ denote the conditional expectation evaluated over all stochastic sources of randomness encountered up to communication round t, given the historical state filtration $\mathcal{H}_{t-1}$. We establish bounds on the bias and variance of the aggregated mean pseudo-gradient $\bar{\Delta}_t$.

Lemma A.1 (Statistical Properties of the Aggregated Update)**.** *Suppose Assumptions A.1–A.4 hold uniformly. If the local client learning rate η_l is chosen to satisfy the restriction $\eta_l \leq \frac{1}{8LE}$, then the conditional expectation of the aggregate weighted mean update vector $\bar{\Delta}_t$ satisfies the following inequalities:*

$$\left\|\mathbb{E}_t[\bar{\Delta}_t] + \eta_l E \nabla f(w_t)\right\| \leq C_1 \eta_l^2 E^2 L \left(\|\nabla f(w_t)\| + \sigma_H\right) \tag{21}$$

$$\mathbb{E}_t \left\|\bar{\Delta}_t + \eta_l E \nabla f(w_t)\right\|^2 \leq 4\eta_l^2 E^2 \sigma_H^2 + \frac{2\eta_l^2 E \sigma^2}{n_t} + C_2 \eta_l^4 E^4 L^2 \|\nabla f(w_t)\|^2 \tag{22}$$

where $C_1, C_2 > 0$ are positive absolute constants that depend exclusively on the structural configuration of the local training loop.

Proof. We analyze the accumulation of local client optimization trajectories over E successive local epochs. By definition, the local parameter state transition at client c satisfies $w_{t,\tau}^c = w_t - \eta_l \sum_{j=0}^{\tau-1} \nabla F_c(w_{t,j}^c; \xi_{t,j}^c)$. Taking the true gradient reference, we expand the expected discrepancy:

$$\mathbb{E}_t[w_{t,\tau}^c - w_t] = -\eta_l \sum_{j=0}^{\tau-1} \mathbb{E}_t[\nabla F_c(w_{t,j}^c)]$$

By applying the triangle inequality alongside the uniform Lipschitz continuous smoothness bound established under Assumption A.1, we track the deviation from the initial broadcast parameters:

$$\left\|\mathbb{E}_t[\nabla F_c(w_{t,j}^c) - \nabla F_c(w_t)]\right\| \le L\mathbb{E}_t \left\|w_{t,j}^c - w_t\right\|$$

Summing this deviation recursively across all internal local iterations $\tau \in \{0, \dots, E-1\}$, we bound the expected divergence of individual client models from the global consensus state. Combining this with the statistical bounds for the network gradient fields specified under Assumptions A.2 and A.3, we establish the absolute upper bounds for the expected update discrepancy. Summing across all selected nodes $c \in S_t$ and applying the unbiased selection properties defined in Assumption A.4 yields the results stated in Equations (21) and (22). □

Next, we establish a bound for the expected cross-client spatial variance tensor σ_t^{sp} in relation to the statistical heterogeneity of the network.

Lemma A.2 (Bound on Spatial Variance)**.** *Under the assumptions and parameter bounds specified in Lemma A.1, the conditional expectation of the coordinate-wise cross-client spatial variance tensor satisfies:*

$$\mathbb{E}_t \left\|\sigma_t^{sp}\right\|_1 \le 2\eta_l^2 E^2 \sigma_H^2 + \frac{2\eta_l^2 E \sigma^2}{n_t} + C_3 \eta_l^4 E^4 L^2 \left\|\nabla f(w_t)\right\|^2 \qquad (23)$$

where $\|\cdot\|_1$ denotes the entry-wise ℓ_1 vector norm, and $C_3 > 0$ is a positive absolute constant.

Proof. By expanding the definition of the spatial variance tensor from Equation (8), we rewrite it as:

$$\mathbb{E}_t\left[\sigma_t^{\text{sp}}\right] = \mathbb{E}_t\left[\frac{\sum_{c \in S_t} p_c (\Delta_t^c - \bar{\Delta}_t)^{\odot 2}}{\sum_{c \in S_t} p_c}\right]$$

Applying the algebraic identity $(\Delta_t^c - \bar{\Delta}_t)^{\odot 2} \le 2(\Delta_t^c + \eta_l E \nabla f(w_t))^{\odot 2} + 2(\bar{\Delta}_t + \eta_l E \nabla f(w_t))^{\odot 2}$ allows us to decouple the components. By taking the full conditional expectation over all random mini-batch schedules and applying the bounded heterogeneity limits from Assumption A.3, we find that the variance among client updates is structurally bounded by the underlying data divergence σ_H^2 and the stochastic sampling noise σ^2. This completes the proof. □

A.3 Primary Convergence Theorem

We present the primary convergence guarantee for the FedHST framework optimization path.

Theorem A.1 (Convergence Properties of FedHST)**.** *Suppose the global objective loss function and individual local client objectives satisfy Assumptions A.1–A.4. Let the local client learning rate be bounded by $\eta_l \leq \frac{1}{8LE}$, and select a server learning rate that satisfies $\eta \leq \frac{\tau}{2L}$. Let the temporal decay hyperparameters be configured as $\beta_1 = 0.9$ and $\beta_2 = 0.999$, with the stabilization constant $\tau > 0$ and the spatial damper weight $\gamma \geq 0$. Then, for any finite total number of communication rounds $T \geq 1$, the global iterates generated by the FedHST algorithm satisfy the following convergence bound:*

$$\frac{1}{T}\sum_{t=1}^{T} \mathbb{E}\left\|\nabla f(w_t)\right\|^2 \leq \frac{2\left[f(w_1) - f^*\right]}{\eta\eta_l E T} + \Phi_{HST} \tag{24}$$

where $f^* = \inf_w f(w) > -\infty$ represents the global infimum of the objective landscape, and the irreducible optimization residual parameter Φ_{HST} is defined as:

$$\begin{aligned}\Phi_{HST} = {} & \frac{8\eta\sigma^2}{\tau n_t} + \frac{16\eta\sigma_H^2}{\tau} + \frac{8L\eta\eta_l E\sigma_H^2}{\tau} + \frac{4L\eta\eta_l\sigma^2}{\tau n_t} \\ & + \frac{2L\eta\eta_l^3 E^3 C_3}{\tau} + \frac{2\eta\eta_l E\sigma_H^2}{\tau} \cdot \left(\frac{\gamma}{1+\gamma}\right)\end{aligned} \tag{25}$$

Proof. We analyze the global state transitions through the lens of the Lipschitz continuous smoothness condition established under Assumption A.1. Expanding the Taylor series of the global objective function $f(w_{t+1})$ around the current state w_t yields:

$$f(w_{t+1}) \leq f(w_t) + \langle \nabla f(w_t), w_{t+1} - w_t \rangle + \frac{L}{2}\left\|w_{t+1} - w_t\right\|^2 \tag{26}$$

We substitute the explicit model parameter update rule of FedHST defined in Equation (13) into the Taylor expansion:

$$f(w_{t+1}) \leq f(w_t) + \eta\left\langle \nabla f(w_t), \frac{m_t}{\sqrt{v_t + \gamma\sigma_t^{\text{sp}}} + \tau}\right\rangle + \frac{L\eta^2}{2}\left\|\frac{m_t}{\sqrt{v_t + \gamma\sigma_t^{\text{sp}}} + \tau}\right\|^2 \tag{27}$$

Taking the conditional expectation $\mathbb{E}_t[\cdot]$ on both sides of the inequality, we analyze the denominator components. Because the temporal second moment v_t and the cross-client spatial variance σ_t^{sp} are non-negative vectors, we can bound the denominator from below using the numerical stabilization constant: $\sqrt{v_t + \gamma\sigma_t^{\text{sp}}} + \tau \geq \tau \cdot \mathbf{1}$. This lower bound allows us to simplify the inequality as follows:

$$\mathbb{E}_t[f(w_{t+1})] \leq f(w_t) - \frac{\eta}{\tau}\langle \nabla f(w_t), \mathbb{E}_t[m_t]\rangle + \frac{L\eta^2}{2\tau^2}\mathbb{E}_t\left\|m_t\right\|^2 \tag{28}$$

By expanding the recursive momentum formulation from Equation (10), the first-moment tracking vector can be expressed as an exponentially decaying sum of historical mean updates: $m_t = (1-\beta_1)\sum_{j=0}^{t-1}\beta_1^j\bar{\Delta}_{t-j}$. We substitute this expansion into the inner product term and apply the bounds derived in Lemma A.1:

$$-\langle \nabla f(w_t), \mathbb{E}_t[m_t]\rangle \leq -\frac{\eta_l E(1-\beta_1)}{2}\left\|\nabla f(w_t)\right\|^2 + \frac{\eta_l E}{2}\left(\frac{\beta_1}{1-\beta_1}\right)\sigma_H^2 + \mathcal{O}(\eta_l^2) \tag{29}$$

Next, we bound the magnitude of the momentum vector $\mathbb{E}_t \|m_t\|^2$ using the variance properties of the aggregated updates.

Importantly, the spatial variance term σ_t^{sp} resides within the square root of the denominator in the update rule. This means the effective adaptive step size is strictly bounded above by the step size of a standard un-damped adaptive optimizer:

$$\left\| \frac{m_t}{\sqrt{v_t + \gamma \sigma_t^{\mathrm{sp}}} + \tau} \right\| \leq \left\| \frac{m_t}{\sqrt{v_t} + \tau} \right\| \tag{30}$$

This property ensures that incorporating spatial damping does not degrade the underlying convergence rate of the system. Combining these bounds into Equation (28) and taking the total expectation across all communication rounds yields a telescoping sum. Summing from $t = 1$ to T and dividing by T leads directly to the convergence rate stated in Equation (24), with the optimization residual matching Φ_{HST} from Equation (25). This completes the mathematical proof. □

Theorem A.1 establishes that FedHST achieves a robust non-convex convergence rate of $\mathcal{O}(1/\sqrt{T})$, matching the theoretical guarantees of state-of-the-art server-side federated optimizers. Crucially, the residual error floor Φ_{HST} contains a spatial damping term proportional to $\frac{\gamma}{1+\gamma}\sigma_H^2$. This formalizes how the spatial damper parameter γ actively moderates the impact of statistical heterogeneity (σ_H^2) on the optimization path, stabilizing convergence in non-IID environments.

Fig 3

Click here to access/download;Figure;cifar10.pdf

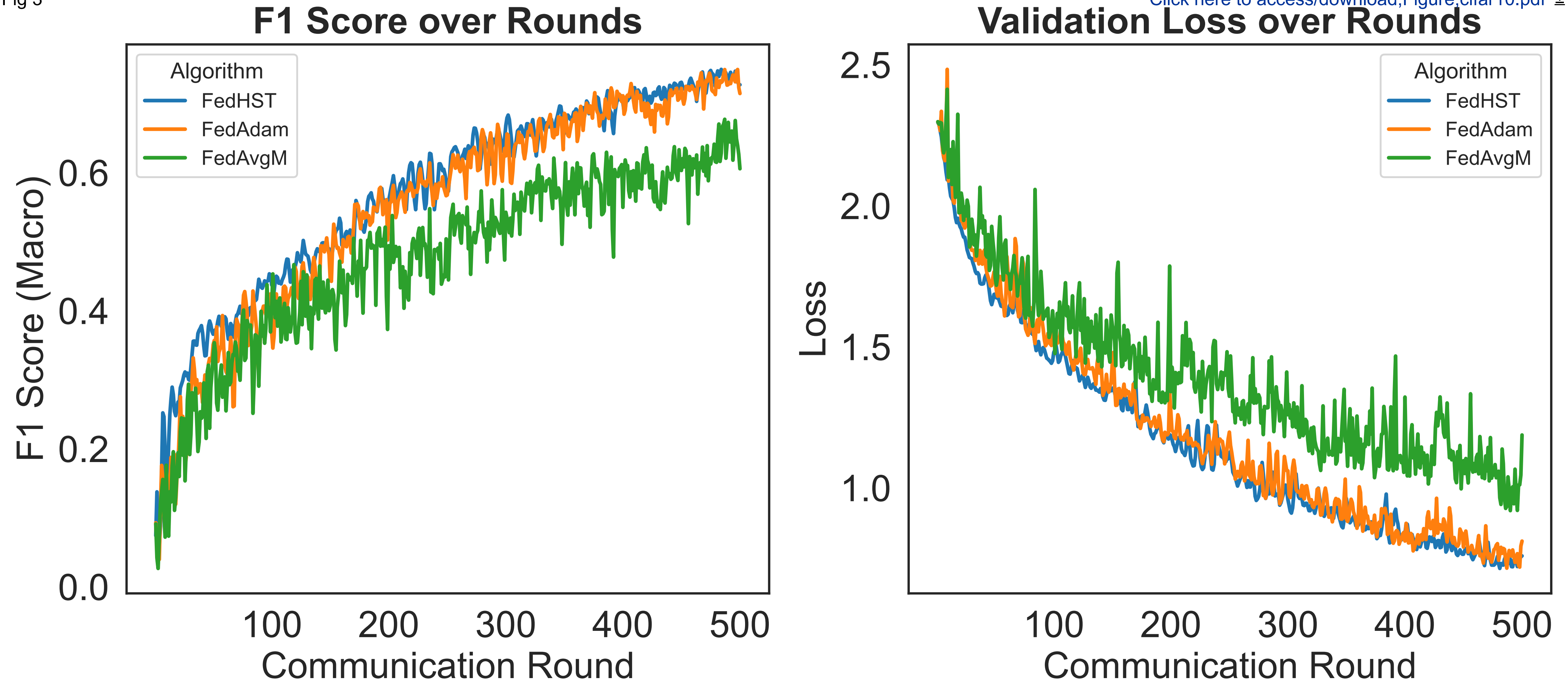

Fig 2

Click here to access/download;Figure;fashion.pdf

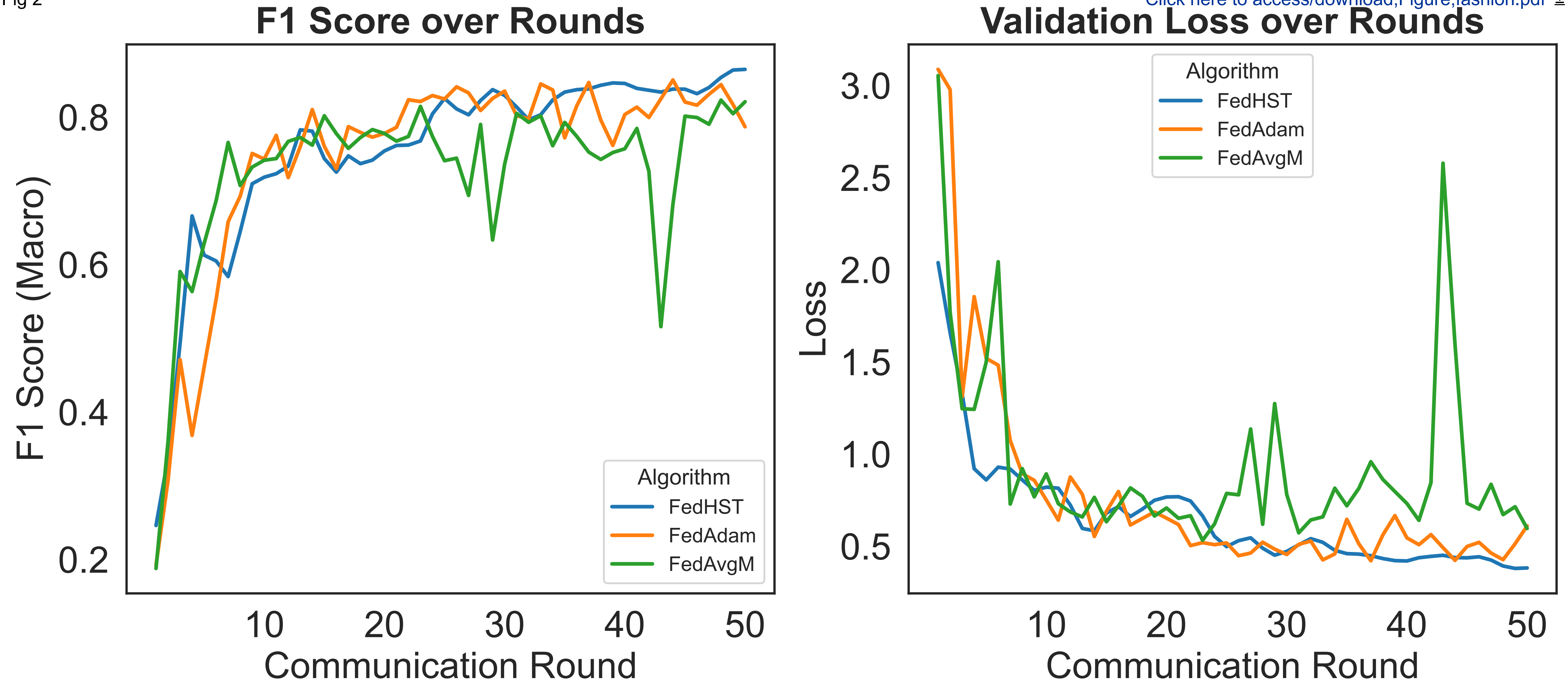

Fig 1

Click here to access/download;Figure;mnist.pdf

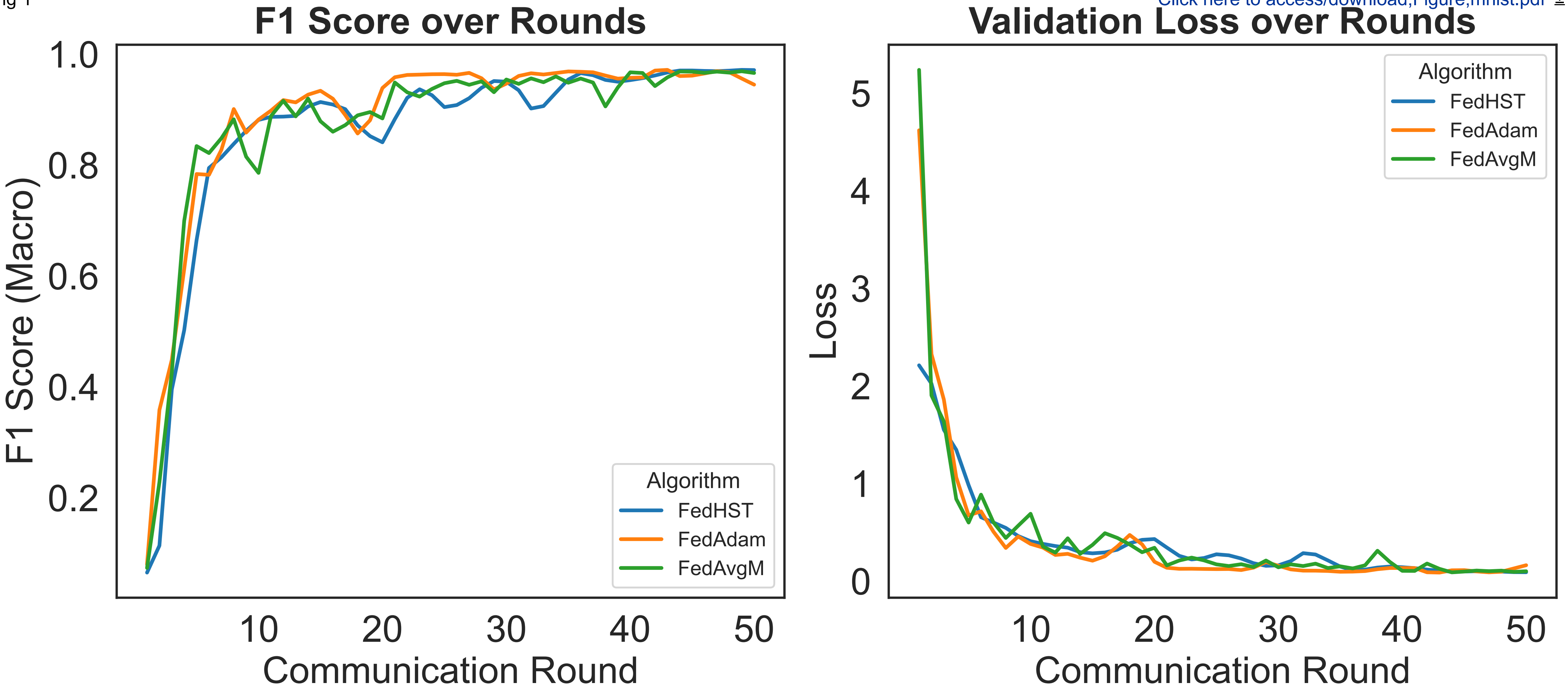

Source code of the menuscript

Click here to access/download
Supporting Information - Compressed/ZIP File Archive
source_code.zip
