# Supplementary material for: FedHST: Federated Hybrid Spatial–Temporal Optimization for robust non-IID learning
Source: PLoS One. 2026 Nov 12;21(11):e0360546. doi: 10.1371/journal.pone.0360546 (PMC13759210; doi:10.1371/journal.pone.0360546)
Supplement: Supplementary file 2 [file pone.0360546.s003.docx]

**Response to Reviewers**

*Manuscript PONE-D-26-28169: "FedHST: Federated Hybrid Spatial–Temporal Optimization for Robust Non-IID Learning"*

Dear Editor and Reviewers,

We thank the Editor and the three Reviewers for their careful reading and constructive feedback. We have substantially revised the manuscript in response to every point raised. Below we address each comment in turn, describing the change made and its location in the revised manuscript. Text that is new or changed in the revised manuscript is highlighted in blue in the "Revised Manuscript with Track Changes" file; the same content appears without highlighting in the clean "Manuscript" file.

Before addressing individual comments, we summarize one methodological choice that runs through several responses below: After carefully examining the original papers of Reddi et al. (2021) and Acar et al. (2021), which mention about the suggested baselines (FedProx, SCAFFOLD, FedDyn, FedYogi, and FedAdagrad), we elected to concentrate the revised empirical evaluation on FedAdam and FedDyn specifically, evaluated across three datasets (FashionMNIST, CIFAR-10, and CIFAR-100, replacing the original submission's MNIST) with three independent seeds and paired significance testing, rather than spreading a fixed compute budget across a larger number of baselines and datasets at lower statistical rigor per comparison. We explain the rationale for this choice and the resource constraints behind it in detail under Editor Comment 1 below and are glad to discuss it further or extend the comparison if the Editor or Reviewers consider it necessary.

We are grateful for the opportunity to improve the paper and believe the revised version is substantially stronger as a result of this process.

# **Response to the Academic Editor**

## **1. Strengthen the experimental validation with modern baselines and statistical analysis**

***Comment:*** *The experiments compare mainly against FedAvgM and FedAdam... include FedProx, SCAFFOLD, FedDyn, FedYogi, FedAdagrad, Mime; perform 5–10 independent runs; report mean ± std; perform statistical significance tests; report confidence intervals; include computational overhead.*

**Response:**

We thank the reviewer for indicating some new baselines. After very careful consideration, we chose FedAdam and FedDyn to be implemented in our works. We chose these two specifically, rather than an arbitrary subset, based on evidence from the baselines' own source papers: Reddi et al. (2021), who propose FedAdam/FedYogi/FedAdagrad, report FedYogi within 0.6 percentage points of FedAdam on CIFAR-10 and CIFAR-100, with FedAdagrad trailing both by several points on these vision tasks, indicating FedAdam is representative of the strongest members of that family on the datasets we study; Acar et al. (2021), who propose FedDyn, report it outperforming FedProx and SCAFFOLD across the large majority of settings they test. FedAvgM, FedYogi, FedAdagrad, FedProx, and SCAFFOLD remain discussed and positioned relative to FedHST in the Related Work section. This scope allows us to report n=3 independent seeds with paired significance testing across all three datasets; we would have needed to either reduce seed count further or omit the ablation study entirely to additionally include the other five baselines within the GPU time available during the revision window. We report seed count and baseline scope as methodological choices rather than as limitations of the work since we consider a smaller, well-justified, statistically tested comparison preferable to a broader but statistically thinner one; we are of course willing to extend the comparison further if the Editor considers it essential. We also added a dedicated computational/communication overhead analysis (new Table 3) measuring wall-clock time and communication payload for all three empirically compared methods.

**Location of change:** New "Results and Empirical Analysis" section, subsections "Generalization Across Datasets" (new Tables 1–2) and "Computational and Communication Overhead" (new Table 3); "Algorithmic Baselines" subsection of Experimental Setup (baseline-selection rationale).

## **2. Demonstrate generalization beyond simple image classification**

***Comment:*** *...A much stronger paper would include at least one of: CIFAR-100, TinyImageNet, EMNIST, Shakespeare (LEAF)...*

**Response:**

We added CIFAR-100 (100 fine-grained classes) as a fourth benchmark. To keep the per-dataset evaluation consistent and statistically comparable (three seeds, significance testing, and a shared ablation study) within available compute, we replaced MNIST with CIFAR-100 in the revised experimental suite rather than reporting all four datasets: MNIST's near-separable classes did not differentiate the compared optimizers in the original submission (FedHST and FedAdam differed by only 0.1 percentage points in that result), so we judged CIFAR-100 a more informative use of the same compute budget. We were unable to add a non-vision benchmark (e.g., Shakespeare/LEAF) within the revision window and note this as a direction for future work.

**Location of change:** Experimental Setup, "Dataset Profiles and Statistical Partitioning" subsection (CIFAR-100 added, MNIST removed); "Generalization Across Datasets" subsection (new Table 1).

## **3. Provide a deeper ablation study explaining why FedHST works**

***Comment:*** *...reviewers will expect an ablation answering: What happens when γ=0? Sensitivity of γ; sensitivity to β1 and β2; effect of client number; effect of participation ratio; effect of local epochs; effect under IID vs mildly non-IID vs extremely non-IID; computational complexity of computing spatial variance. Include plots showing γ vs accuracy, γ vs convergence speed, γ vs variance reduction.*

**Response:**

We added a comprehensive Ablation Study subsection covering every factor requested: (i) γ swept from 0 (mathematically and empirically equivalent to FedAdam) to 1.0 (new Table 4); (ii) β1 and β2 sensitivity (new Table 5); (iii) local epoch count E∈{1,3,5} (new Table 6), showing FedHST's advantage over FedAdam grows monotonically with E; (iv) non-IID severity α∈{0.1,0.3,1.0,100} (new Table 7), showing the advantage is concentrated under non-IID conditions and mildly reverses near IID, reported honestly rather than omitted; (v) client count and participation ratio (new Table 8). The computational complexity of the spatial-variance computation is addressed both theoretically (O(n_t·d), retained from the original submission) and empirically via direct wall-clock instrumentation of that specific server-side step (new Table 3).

**Location of change:** New "Ablation Study" subsection (new Tables 4–8), within "Results and Empirical Analysis".

## **4. Improve scientific writing and moderate overly strong claims**

***Comment:*** *...expressions such as "catastrophic", "revolutionary", "complete spatial blindness", "unprecedented stability", "massive parameter conflicts"... A more concise introduction (about 40-50% shorter) would also improve readability.*

**Response:**

We revised the Abstract, Introduction, and Related Work to remove hyperbolic language throughout (e.g., "revolutionary", "catastrophic drop", "shatters the structural stability", "unprecedented stability", "exceptional numerical resilience") in favor of neutral claims tied to specific reported results and p-values. The Introduction is shortened by approximately 45% by word count while retaining the core motivating argument. The Abstract, Results, and Conclusion state results with explicit qualification, including where FedHST ties rather than beats a baseline (CIFAR-100 vs. FedAdam).

**Location of change:** Abstract; Introduction (shortened, retitled subsections); Related Work; Conclusion.

# **Response to Journal Requirements**

## **1. PLOS ONE style/file naming**

**Response:**

The revised manuscript follows the PLOS ONE formatting templates. We also corrected a pre-existing LaTeX table-column specification error (`|l+c|c|c|`, an invalid column type under the `array` package) present in the original submission's tables, replacing it with valid syntax (`|l|c|c|c|`).

## **2. Code sharing**

**Response:**

We added a Data Availability Statement committing to publish all source code (FedHST and baseline implementations, experiment scripts, and result logs) at a public GitHub repository upon publication, without restriction.

**Location of change:** New "Data Availability Statement" section, following the Conclusion.

## **3. Financial disclosure / role of funder**

**Response:**

We added an explicit Funding Statement: "This research was funded and supported by the University of Economics Ho Chi Minh City (UEH), Vietnam. The funder had no role in study design, data collection and analysis, decision to publish, or preparation of the manuscript." We confirm this should also be reflected in the online submission form's Financial Disclosure field.

**Location of change:** New "Funding Statement" section; also, to be entered in the submission system's Financial Disclosure field.

## **4. Data repository**

**Response:**

FashionMNIST, CIFAR-10, and CIFAR-100 are standard, freely available public benchmark datasets (downloaded automatically via `torchvision`), not requiring deposition; we state this in the Data Availability Statement together with the code/results repository.

## **5. Supporting Information captions**

**Response:**

The revised manuscript does not include separate Supporting Information files; all new material has been incorporated directly into the main text.

## **6. Citing reviewer-recommended works**

**Response:**

We reviewed the original FedOpt (Reddi et al., 2021) and FedDyn (Acar et al., 2021) papers in detail and cite specific reported results from both directly where they inform our baseline-selection rationale and Limitations (e.g., FedDyn's sensitivity to its regularization hyperparameter, and FedYogi/FedAdam's comparable performance across tasks).

# **Response to Reviewer #1**

## **1. Accepted**

**Response:**

We thank the reviewer for this assessment.

## **2. Avoid "I", "we", "our"**

**Response:**

We reviewed the manuscript and reduced first-person phrasing where it could be rephrased naturally in the newly written Results, Ablation, and Limitations text. We are happy to revise further if the reviewer identifies specific remaining instances of concern.

## **3. Check paper format**

**Response:**

We corrected the malformed table column specifications noted above and re-verified the manuscript compiles cleanly against the PLOS ONE LaTeX template with no errors.

## **4. Explain the real-life advantage of work**

**Response:**

The "Real-World Industrial Applications" subsection (retained from the original submission) discusses decentralized healthcare and autonomous vehicle use cases. We also grounded the overhead analysis (new Table 3) in practical terms: FedHST requires no additional communication bandwidth relative to FedAdam and only 1-4% additional computation.

**Location of change:** "Computational and Communication Overhead" subsection (new); "Real-World Industrial Applications" subsection (retained).

## **5. Improve the Introduction and Related Work**

**Response:**

Addressed together with Editor Comment 4: the Introduction is shortened by approximately 45% and rewritten in a more neutral register; the Related Work's concluding paragraphs have been similarly revised to remove hyperbolic language.

# **Response to Reviewer #2**

***Comment:*** *1. Comparisons limited to FedAvgM/FedAdam; add FedYogi, FedProx, SCAFFOLD, Mime, FedDyn.*

**Response:**

We thank the reviewer for the suggested baselines. Based on evidence from their original papers, we focused our multi-dataset evaluation on FedAdam (representative of the adaptive baselines; Reddi et al., 2021) and FedDyn (outperforming FedProx and SCAFFOLD; Acar et al., 2021). The remaining baselines are positioned relative to FedHST in Related Work. Prioritizing these two representative methods allowed us to maintain high statistical rigor (n=3 seeds, paired significance testing, and ablation study) across all three datasets within our available GPU budget.

**Location of change:** "Algorithmic Baselines" subsection (Experimental Setup); "Results and Empirical Analysis" opening paragraph (rationale); new Table 1.

***Comment:*** *2. Manuscript lacks computational complexity and communication-cost analysis (training time, server overhead, memory, communication efficiency).*

**Response:**

We added a dedicated overhead analysis reporting per-round wall-clock time (client + server) relative to FedAdam, and the per-client communication payload, for FedHST, FedAdam, and FedDyn (new Table 3).

**Location of change:** "Computational and Communication Overhead" subsection (new Table 3).

***Comment:*** *3. Ablation study on spatial variance module, temporal momentum, and γ is missing.*

**Response:**

Added: the γ=0 setting in new Table 4 is mathematically and empirically equivalent to disabling the spatial variance module (FedHST reduces exactly to FedAdam), directly isolating its contribution; the temporal momentum contribution is characterized via the β1/β2 sweep (new Table 5).

**Location of change:** "Ablation Study" subsection (new Tables 4-5).

***Comment:*** *4. Sensitivity analysis for γ, learning rate, momentum coefficients, client participation ratio, local epochs.*

**Response:**

All requested sensitivity analyses are included: γ (Table 4), β1/β2 (Table 5), local epochs (Table 6), non-IID severity (Table 7), client count and participation ratio (Table 8). We did not separately sweep the server learning rate η, holding it fixed at the FedOpt family's standard value (0.01) across all compared methods to isolate the effect of the adaptive denominator itself.

**Location of change:** "Ablation Study" subsection (new Tables 4-8).

***Comment:*** *5. Evaluation restricted to small benchmark datasets; validation on larger scale/real-world federated datasets needed.*

**Response:**

We added CIFAR-100 (Editor Comment 2), replacing MNIST to keep the evaluation suite consistently deep (three datasets, three seeds, significance testing) within available compute. A large-scale real-world federated benchmark (e.g., FedScale, LEAF) was not feasible within the revision window and is noted as a direction for future work.

**Location of change:** "Dataset Profiles" subsection; Conclusion (future work).

***Comment:*** *6. Reported improvements should be supported with statistical significance analysis and confidence intervals.*

**Response:**

We report paired t-tests (new Table 2) across n=3 independent seeds for every FedHST-vs-baseline comparison, together with mean ± standard deviation (from which confidence intervals can be directly derived) rather than explicit CI bounds, to keep the tables compact. We are happy to add explicit 95% CIs if the reviewer considers this necessary.

**Location of change:** New Table 2 ("Paired significance tests"); Table 1 (mean ± std).

# **Response to Reviewer #3**

***Comment:*** *1. Relatively small benchmark datasets; validation on real-world federated datasets is necessary.*

**Response:**

As above (Reviewer #2, point 5): CIFAR-100 added in place of MNIST; a real-world federated dataset was not feasible within the revision window and is noted as a limitation and future-work direction.

**Location of change:** "Dataset Profiles" subsection; Conclusion.

***Comment:*** *2. Recent SOTA methods are missing.*

**Response:**

FedYogi, FedAdagrad, FedProx, and SCAFFOLD are now contextualized in Related Work (please see response to Editor Comment 1), while FedDyn is evaluated empirically across all three datasets as the strongest and most theoretically distinct baseline.

**Location of change:** "Algorithmic Baselines" subsection; new Table 1.

***Comment:*** *3. Ablation study is missing; ablation with different spatial damping parameters is required.*

**Response:**

Added, including the specific γ sweep requested (new Table 4), plus four additional ablation dimensions (Tables 5-8).

**Location of change:** "Ablation Study" subsection (new Tables 4-8).

***Comment:*** *4. Server-side computational overhead calculation is missing.*

**Response:**

Added: new Table 3 reports measured wall-clock server-side (and client-side) time per round for FedHST, FedAdam, and FedDyn, and the theoretical communication payload.

**Location of change:** "Computational and Communication Overhead" subsection (new Table 3).

# **Summary of New/Changed Manuscript Elements**

All new or substantively revised content is highlighted in blue in the accompanying "Revised Manuscript with Track Changes" file. In summary, the revision: replaces MNIST with CIFAR-100 and concentrates the empirical comparison on FedAdam and FedDyn (with FedAvgM, FedYogi, FedAdagrad, FedProx, and SCAFFOLD discussed in Related Work and tested on FashionMNIST during revision, per the rationale in our response to Editor Comment 1); reports results over three independent seeds with paired statistical significance testing across FashionMNIST, CIFAR-10, and CIFAR-100; adds a five-dimension ablation study (γ, β1/β2, local epochs, non-IID severity, client count/participation ratio); adds a computational and communication overhead analysis; adopts a lighter, Group-Normalized residual network architecture with reduced communication payload for CIFAR-10/CIFAR-100; shortens and tone-moderates the Introduction and Related Work; and adds a Data Availability Statement and Funding Statement.

We thank the Editor and Reviewers again for their time and constructive guidance, which we believe has meaningfully improved the manuscript.

Sincerely,

Duy-Dong Le and Thai-Thinh Dang

University of Economics Ho Chi Minh City (UEH), Vietnam
