# Supplementary material for: FedHST: Federated Hybrid Spatial–Temporal Optimization for robust non-IID learning
Source: PLoS One. 2026 Nov 12;21(11):e0360546. doi: 10.1371/journal.pone.0360546 (PMC13759210; doi:10.1371/journal.pone.0360546)
Supplement: Peer review history

# Peer review: Decision Letter 0

**DOI:** 10.1371/journal.pone.0360546.r001

**Authors:** Debotosh Bhattacharjee

31 Jul 2026

Dear Dr. Le,

Thank you for submitting your manuscript to PLOS ONE. After careful consideration, we feel that it has merit but does not fully meet PLOS ONE’s publication criteria as it currently stands. Therefore, we invite you to submit a revised version of the manuscript that addresses the points raised during the review process.

==============================

**Please revise your manuscript as per reviewers' and editor's comments.**

==============================

Please submit your revised manuscript by Sep 29 2026 11:59PM. If you will need more time than this to complete your revisions, please reply to this message or contact the journal office at plosone@plos.org. When you're ready to submit your revision, log on to https://www.editorialmanager.com/pone/ and select the 'Submissions Needing Revision' folder to locate your manuscript file.

- A letter that responds to each point raised by the academic editor and reviewer(s). You should upload this letter as a separate file labeled 'Response to Reviewers'.
- A marked-up copy of your manuscript that highlights changes made to the original version. You should upload this as a separate file labeled 'Revised Manuscript with Track Changes'.
- An unmarked version of your revised paper without tracked changes. You should upload this as a separate file labeled 'Manuscript'.

If you would like to make changes to your financial disclosure, please include your updated statement in your cover letter. Guidelines for resubmitting your figure files are available below the reviewer comments at the end of this letter.

If applicable, we recommend that you deposit your laboratory protocols in protocols.io to enhance the reproducibility of your results. Protocols.io assigns your protocol its own identifier (DOI) so that it can be cited independently in the future. For instructions see: https://journals.plos.org/plosone/s/submission-guidelines#loc-laboratory-protocols. Additionally, PLOS ONE offers an option for publishing peer-reviewed Lab Protocol articles, which describe protocols hosted on protocols.io. Read more information on sharing protocols at https://plos.org/protocols?utm_medium=editorial-email&utm_source=authorletters&utm_campaign=protocols.

As the corresponding author, your ORCID iD is verified in the submission system and will appear in the published article. PLOS supports the use of ORCID, and we encourage all coauthors to register for an ORCID iD and use it as well. Please encourage your coauthors to verify their ORCID iD within the submission system before final acceptance, as unverified ORCID iDs will not appear in the published article. *Only*  the individual author can complete the verification step; PLOS staff *cannot*  verify ORCID iDs on behalf of authors.

We look forward to receiving your revised manuscript.

Kind regards,

Debotosh Bhattacharjee, PhD

Academic Editor

PLOS One

Journal Requirements:

When submitting your revision, we need you to address these additional requirements.

1.Please ensure that your manuscript meets PLOS ONE's style requirements, including those for file naming. The PLOS ONE style templates can be found at

https://journals.plos.org/plosone/s/file?id=wjVg/PLOSOne_formatting_sample_main_body.pdf and

https://journals.plos.org/plosone/s/file?id=ba62/PLOSOne_formatting_sample_title_authors_affiliations.pdf

2. Please note that PLOS One has specific guidelines on code sharing for submissions in which author-generated code underpins the findings in the manuscript. In these cases, we expect all author-generated code to be made available without restrictions upon publication of the work. Please review our guidelines at https://journals.plos.org/plosone/s/materials-and-software-sharing#loc-sharing-code and ensure that your code is shared in a way that follows best practice and facilitates reproducibility and reuse.

3. Thank you for stating the following financial disclosure:

“This research is funded and supported by the University of Economics Ho Chi Minh City (UEH), Vietnam.”

Please state what role the funders took in the study.  If the funders had no role, please state: "The funders had no role in study design, data collection and analysis, decision to publish, or preparation of the manuscript."

If this statement is not correct you must amend it as needed.

Please include this amended Role of Funder statement in your cover letter; we will change the online submission form on your behalf.

4. Thank you for uploading your study's underlying data set. Unfortunately, the repository you have noted in your Data Availability statement does not qualify as an acceptable data repository according to PLOS's standards.

At this time, please upload the minimal data set necessary to replicate your study's findings to a stable, public repository (such as figshare or Dryad) and provide us with the relevant URLs, DOIs, or accession numbers that may be used to access these data. For a list of recommended repositories and additional information on PLOS standards for data deposition, please see https://journals.plos.org/plosone/s/recommended-repositories.

5. Please include captions for your Supporting Information files at the end of your manuscript, and update any in-text citations to match accordingly. Please see our Supporting Information guidelines for more information: http://journals.plos.org/plosone/s/supporting-information.

6. If the reviewer comments include a recommendation to cite specific previously published works, please review and evaluate these publications to determine whether they are relevant and should be cited. There is no requirement to cite these works unless the editor has indicated otherwise.

Additional Editor Comments:

This paper has a promising core idea—a hybrid server-side optimizer combining temporal moments with cross-client spatial variance—but it still has several weaknesses that PLOS ONE reviewers already raised. The manuscript proposes a new optimization framework, derives a non-convex convergence result, and evaluates it on MNIST, FashionMNIST, and CIFAR-10 under non-IID settings. The authors are requested to revise the paper as per suggestions from the reviewers and following:

1. Strengthen the experimental validation with modern baselines and statistical analysis

This is the biggest weakness.

The experiments compare mainly against FedAvgM and FedAdam, whereas the related work discusses many stronger and more recent federated optimization methods such as FedProx, SCAFFOLD, FedDyn, FedYogi, Mime, and others.

To satisfy PLOS ONE reviewers, include:

• FedProx

• SCAFFOLD

• FedDyn

• FedYogi

• FedAdagrad

• Mime (if computationally feasible)

Additionally,

• perform at least 5–10 independent runs

• report mean ± standard deviation

• perform statistical significance tests (Wilcoxon or paired t-test)

• report confidence intervals

• include computational overhead (training time and server computation)

2. Demonstrate generalization beyond simple image classification

Currently, experiments are limited to

• MNIST

• FashionMNIST

• CIFAR-10

These are relatively small benchmark datasets and may not convince reviewers that the optimizer is broadly useful.

A much stronger paper would include at least one of:

• CIFAR-100

• TinyImageNet

• EMNIST

• Shakespeare (LEAF)

• Sent140

• StackOverflow

• medical FL benchmark

• real federated benchmark (LEAF/FedScale)

3. Provide a deeper ablation study explaining why FedHST works

The paper introduces

• spatial variance

• temporal variance

• hybrid denominator

• damping coefficient γ

which is the novelty of the method.

However, reviewers will expect an ablation answering questions such as:

• What happens when γ = 0?

• Sensitivity of γ

• Sensitivity to β₁ and β₂

• Effect of client number

• Effect of participation ratio

• Effect of local epochs

• Effect under IID vs mildly non-IID vs extremely non-IID

• Computational complexity of computing spatial variance

Include plots showing:

• γ versus accuracy

• γ versus convergence speed

• γ versus variance reduction

4. Improve the scientific writing and moderate overly strong claims

This is probably the largest presentation issue.

The manuscript repeatedly uses expressions such as

• "catastrophic"

• "revolutionary"

• "complete spatial blindness"

• "unprecedented stability"

• "massive parameter conflicts"

throughout the Introduction and Related Work.

PLOS ONE reviewers generally expect a neutral scientific tone.

Examples:

Instead of

"FedHST achieves unprecedented stability."

write

"FedHST consistently improves convergence stability under the evaluated non-IID settings."

Instead of

"complete spatial blindness"

write

"existing methods do not explicitly model intra-round cross-client variance."

Likewise, avoid implying that existing methods are fundamentally flawed unless this is rigorously demonstrated experimentally.

A more concise introduction (about 40–50% shorter) would also improve readability.

[Note: HTML markup is below. Please do not edit.]

Reviewer's Responses to Questions

**Comments to the Author**

1. Is the manuscript technically sound, and do the data support the conclusions?

Reviewer #1: Yes

Reviewer #2: Partly

Reviewer #3: Partly

**********

2. Has the statistical analysis been performed appropriately and rigorously?

Reviewer #1: Yes

Reviewer #2: No

Reviewer #3: No

**********

3. Have the authors made all data underlying the findings in their manuscript fully available?

The PLOS Data policy

Reviewer #1: Yes

Reviewer #2: No

Reviewer #3: No

**********

4. Is the manuscript presented in an intelligible fashion and written in standard English?

Reviewer #1: Yes

Reviewer #2: Yes

Reviewer #3: Yes

**********

Reviewer #1: 1. Accepted

2. Don't use the I, We, our this types of word .

3.Please check the paper format.

4. You should explain the real life advantage of your work properly.

5. You should write the introduction and related work properly.

Reviewer #2: 1. The experimental comparison is limited to FedAvgM and FedAdam. Comparisons with additional state-of-the-art federated optimization methods such as FedYogi, FedProx, SCAFFOLD, Mime, and FedDyn would provide a more comprehensive evaluation.

2. The manuscript lacks computational complexity and communication-cost analysis. Training time, server computation overhead, memory consumption, and communication efficiency should be quantitatively reported.

3. An ablation study evaluating the individual contributions of the spatial variance module, temporal momentum, and the spatial damper weight (γ) is missing and should be included.

4. Sensitivity analysis for important hyperparameters, including γ, learning rate, momentum coefficients, client participation ratio, and local training epochs, would strengthen the experimental validation.

5. The evaluation is restricted to relatively small benchmark datasets. Validation on larger-scale and real-world federated datasets would better demonstrate the scalability and practical applicability of the proposed method.

6. The reported performance improvements should be supported with statistical significance analysis (e.g., paired t-test or Wilcoxon signed-rank test) and confidence intervals to confirm that the observed gains are statistically meaningful.

Reviewer #3: The paper presents a novel hybrid spatial–temporal optimization strategy to overcome the limitation of existing server-side federated optimizers. The proposed FedHST is well-motivated. The mathamatical proof of non-convex convergence analysis also justify it. Experimental results on multiple benchmark datasets under severe non-IID settings demonstrates consistent performance. The convergence stability and predictive performance over strong baselines also shows the superiority.

However, the major concerns which lead to major revisions of the paper are as follows:

1. Relatively small benchmark datasets are used. Validation on real-world federated datasets are necessary.

2. Recent SOTA methds are missing.

3. Ablation study is missing. Ablation study with different spatial damping parameter is required.

4. Server side computational overhead calculation is missing.

**********

what does this mean?). If published, this will include your full peer review and any attached files.

If you choose “no”, your identity will remain anonymous but your review may still be made public.

**Do you want your identity to be public for this peer review?**  For information about this choice, including consent withdrawal, please see our Privacy Policy

Reviewer #1: No

Reviewer #2: No

Reviewer #3: **Yes:**  Hiranmoy Roy

**********

[NOTE: If reviewer comments were submitted as an attachment file, they will be attached to this email and accessible via the submission site. Please log into your account, locate the manuscript record, and check for the action link "View Attachments". If this link does not appear, there are no attachment files.]

To ensure your figures meet our technical requirements, please review our figure guidelines: https://journals.plos.org/plosone/s/figures

You may also use PLOS’s free figure tool, NAAS, to help you prepare publication quality figures: https://journals.plos.org/plosone/s/figures#loc-tools-for-figure-preparation.

NAAS will assess whether your figures meet our technical requirements by comparing each figure against our figure specifications.

Attachment Submitted filename: PONE-D-26-28169.pdf

# Author comment: Author response to Decision Letter 1

**DOI:** 10.1371/journal.pone.0360546.r002

2 Sep 2026

Response to Reviewers

Manuscript PONE-D-26-28169: "FedHST: Federated Hybrid Spatial–Temporal Optimization for Robust Non-IID Learning"

Dear Editor and Reviewers,

We thank the Editor and the three Reviewers for their careful reading and constructive feedback. We have substantially revised the manuscript in response to every point raised. Below we address each comment in turn, describing the change made and its location in the revised manuscript. Text that is new or changed in the revised manuscript is highlighted in blue in the "Revised Manuscript with Track Changes" file; the same content appears without highlighting in the clean "Manuscript" file.

Before addressing individual comments, we summarize one methodological choice that runs through several responses below: After carefully examining the original papers of Reddi et al. (2021) and Acar et al. (2021), which mention about the suggested baselines (FedProx, SCAFFOLD, FedDyn, FedYogi, and FedAdagrad), we elected to concentrate the revised empirical evaluation on FedAdam and FedDyn specifically, evaluated across three datasets (FashionMNIST, CIFAR-10, and CIFAR-100, replacing the original submission's MNIST) with three independent seeds and paired significance testing, rather than spreading a fixed compute budget across a larger number of baselines and datasets at lower statistical rigor per comparison. We explain the rationale for this choice and the resource constraints behind it in detail under Editor Comment 1 below and are glad to discuss it further or extend the comparison if the Editor or Reviewers consider it necessary.

We are grateful for the opportunity to improve the paper and believe the revised version is substantially stronger as a result of this process.

Response to the Academic Editor

1. Strengthen the experimental validation with modern baselines and statistical analysis

Comment: The experiments compare mainly against FedAvgM and FedAdam... include FedProx, SCAFFOLD, FedDyn, FedYogi, FedAdagrad, Mime; perform 5–10 independent runs; report mean ± std; perform statistical significance tests; report confidence intervals; include computational overhead.

Response:

We thank the reviewer for indicating some new baselines. After very careful consideration, we chose FedAdam and FedDyn to be implemented in our works. We chose these two specifically, rather than an arbitrary subset, based on evidence from the baselines' own source papers: Reddi et al. (2021), who propose FedAdam/FedYogi/FedAdagrad, report FedYogi within 0.6 percentage points of FedAdam on CIFAR-10 and CIFAR-100, with FedAdagrad trailing both by several points on these vision tasks, indicating FedAdam is representative of the strongest members of that family on the datasets we study; Acar et al. (2021), who propose FedDyn, report it outperforming FedProx and SCAFFOLD across the large majority of settings they test. FedAvgM, FedYogi, FedAdagrad, FedProx, and SCAFFOLD remain discussed and positioned relative to FedHST in the Related Work section. This scope allows us to report n=3 independent seeds with paired significance testing across all three datasets; we would have needed to either reduce seed count further or omit the ablation study entirely to additionally include the other five baselines within the GPU time available during the revision window. We report seed count and baseline scope as methodological choices rather than as limitations of the work since we consider a smaller, well-justified, statistically tested comparison preferable to a broader but statistically thinner one; we are of course willing to extend the comparison further if the Editor considers it essential. We also added a dedicated computational/communication overhead analysis (new Table 3) measuring wall-clock time and communication payload for all three empirically compared methods.

Location of change: New "Results and Empirical Analysis" section, subsections "Generalization Across Datasets" (new Tables 1–2) and "Computational and Communication Overhead" (new Table 3); "Algorithmic Baselines" subsection of Experimental Setup (baseline-selection rationale).

2. Demonstrate generalization beyond simple image classification

Comment: ...A much stronger paper would include at least one of: CIFAR-100, TinyImageNet, EMNIST, Shakespeare (LEAF)...

Response:

We added CIFAR-100 (100 fine-grained classes) as a fourth benchmark. To keep the per-dataset evaluation consistent and statistically comparable (three seeds, significance testing, and a shared ablation study) within available compute, we replaced MNIST with CIFAR-100 in the revised experimental suite rather than reporting all four datasets: MNIST's near-separable classes did not differentiate the compared optimizers in the original submission (FedHST and FedAdam differed by only 0.1 percentage points in that result), so we judged CIFAR-100 a more informative use of the same compute budget. We were unable to add a non-vision benchmark (e.g., Shakespeare/LEAF) within the revision window and note this as a direction for future work.

Location of change: Experimental Setup, "Dataset Profiles and Statistical Partitioning" subsection (CIFAR-100 added, MNIST removed); "Generalization Across Datasets" subsection (new Table 1).

3. Provide a deeper ablation study explaining why FedHST works

Comment: ...reviewers will expect an ablation answering: What happens when γ=0? Sensitivity of γ; sensitivity to β1 and β2; effect of client number; effect of participation ratio; effect of local epochs; effect under IID vs mildly non-IID vs extremely non-IID; computational complexity of computing spatial variance. Include plots showing γ vs accuracy, γ vs convergence speed, γ vs variance reduction.

Response:

We added a comprehensive Ablation Study subsection covering every factor requested: (i) γ swept from 0 (mathematically and empirically equivalent to FedAdam) to 1.0 (new Table 4); (ii) β1 and β2 sensitivity (new Table 5); (iii) local epoch count E∈{1,3,5} (new Table 6), showing FedHST's advantage over FedAdam grows monotonically with E; (iv) non-IID severity α∈{0.1,0.3,1.0,100} (new Table 7), showing the advantage is concentrated under non-IID conditions and mildly reverses near IID, reported honestly rather than omitted; (v) client count and participation ratio (new Table 8). The computational complexity of the spatial-variance computation is addressed both theoretically (O(n_t·d), retained from the original submission) and empirically via direct wall-clock instrumentation of that specific server-side step (new Table 3).

Location of change: New "Ablation Study" subsection (new Tables 4–8), within "Results and Empirical Analysis".

4. Improve scientific writing and moderate overly strong claims

Comment: ...expressions such as "catastrophic", "revolutionary", "complete spatial blindness", "unprecedented stability", "massive parameter conflicts"... A more concise introduction (about 40-50% shorter) would also improve readability.

Response:

We revised the Abstract, Introduction, and Related Work to remove hyperbolic language throughout (e.g., "revolutionary", "catastrophic drop", "shatters the structural stability", "unprecedented stability", "exceptional numerical resilience") in favor of neutral claims tied to specific reported results and p-values. The Introduction is shortened by approximately 45% by word count while retaining the core motivating argument. The Abstract, Results, and Conclusion state results with explicit qualification, including where FedHST ties rather than beats a baseline (CIFAR-100 vs. FedAdam).

Location of change: Abstract; Introduction (shortened, retitled subsections); Related Work; Conclusion.

Response to Journal Requirements

1. PLOS ONE style/file naming

Response:

The revised manuscript follows the PLOS ONE formatting templates. We also corrected a pre-existing LaTeX table-column specification error (`|l+c|c|c|`, an invalid column type under the `array` package) present in the original submission's tables, replacing it with valid syntax (`|l|c|c|c|`).

2. Code sharing

Response:

We added a Data Availability Statement committing to publish all source code (FedHST and baseline implementations, experiment scripts, and result logs) at a public GitHub repository upon publication, without restriction.

Location of change: New "Data Availability Statement" section, following the Conclusion.

3. Financial disclosure / role of funder

Response:

We added an explicit Funding Statement: "This research was funded and supported by the University of Economics Ho Chi Minh City (UEH), Vietnam. The funder had no role in study design, data collection and analysis, decision to publish, or preparation of the manuscript." We confirm this should also be reflected in the online submission form's Financial Disclosure field.

Location of change: New "Funding Statement" section; also, to be entered in the submission system's Financial Disclosure field.

4. Data repository

Response:

FashionMNIST, CIFAR-10, and CIFAR-100 are standard, freely available public benchmark datasets (downloaded automatically via `torchvision`), not requiring deposition; we state this in the Data Availability Statement together with the code/results repository.

5. Supporting Information captions

Response:

The revised manuscript does not include separate Supporting Information files; all new material has been incorporated directly into the main text.

6. Citing reviewer-recommended works

Response:

We reviewed the original FedOpt (Reddi et al., 2021) and FedDyn (Acar et al., 2021) papers in detail and cite specific reported results from both directly where they inform our baseline-selection rationale and Limitations (e.g., FedDyn's sensitivity to its regularization hyperparameter, and FedYogi/FedAdam's comparable performance across tasks).

Response to Reviewer #1

1. Accepted

Response:

We thank the reviewer for this assessment.

2. Avoid "I", "we", "our"

Response:

We reviewed the manuscript and reduced first-person phrasing where it could be rephrased naturally in the newly written Results, Ablation, and Limitations text. We are happy to revise further if the reviewer identifies specific remaining instances of concern.

3. Check paper format

Response:

We corrected the malformed table column specifications noted above and re-verified the manuscript compiles cleanly against the PLOS ONE LaTeX template with no errors.

4. Explain the real-life advantage of work

Response:

The "Real-World Industrial Applications" subsection (retained from the original submission) discusses decentralized healthcare and autonomous vehicle use cases. We also grounded the overhead analysis (new Table 3) in practical terms: FedHST requires no additional communication bandwidth relative to FedAdam and only 1-4% additional computation.

Location of change: "Computational and Communication Overhead" subsection (new); "Real-World Industrial Applications" subsection (retained).

5. Improve the Introduction and Related Work

Response:

Addressed together with Editor Comment 4: the Introduction is shortened by approximately 45% and rewritten in a more neutral register; the Related Work's concluding paragraphs have been similarly revised to remove hyperbolic language.

Response to Reviewer #2

Comment: 1. Comparisons limited to FedAvgM/FedAdam; add FedYogi, FedProx, SCAFFOLD, Mime, FedDyn.

Response:

We thank the reviewer for the suggested baselines. Based on evidence from their original papers, we focused our multi-dataset evaluation on FedAdam (representative of the adaptive baselines; Reddi et al., 2021) and FedDyn (outperforming FedProx and SCAFFOLD; Acar et al., 2021). The remaining baselines are positioned relative to FedHST in Related Work. Prioritizing these two representative methods allowed us to maintain high statistical rigor (n=3 seeds, paired significance testing, and ablation study) across all three datasets within our available GPU budget.

Location of change: "Algorithmic Baselines" subsection (Experimental Setup); "Results and Empirical Analysis" opening paragraph (rationale); new Table 1.

Comment: 2. Manuscript lacks computational complexity and communication-cost analysis (training time, server overhead, memory, communication efficiency).

Response:

We added a dedicated overhead analysis reporting per-round wall-clock time (client + server) relative to FedAdam, and the per-client communication payload, for FedHST, FedAdam, and FedDyn (new Table 3).

Location of change: "Computational and Communication Overhead" subsection (new Table 3).

Comment: 3. Ablation study on spatial variance module, temporal momentum, and γ is missing.

Response:

Added: the γ=0 setting in new Table 4 is mathematically and empirically equivalent to disabling the spatial variance module (FedHST reduces exactly to FedAdam), directly isolating its contribution; the temporal momentum contribution is characterized via the β1/β2 sweep (new Table 5).

Location of change: "Ablation Study" subsection (new Tables 4-5).

Comment: 4. Sensitivity analysis for γ, learning rate, momentum coefficients, client participation ratio, local epochs.

Response:

All requested sensitivity analyses are included: γ (Table 4), β1/β2 (Table 5), local epochs (Table 6), non-IID severity (Table 7), client count and participation ratio (Table 8). We did not separately sweep the server learning rate η, holding it fixed at the FedOpt family's standard value (0.01) across all compared methods to isolate the effect of the adaptive denominator itself.

Location of change: "Ablation Study" subsection (new Tables 4-8).

Comment: 5. Evaluation restricted to small benchmark datasets; validation on larger scale/real-world federated datasets needed.

Response:

We added CIFAR-100 (Editor Comment 2), replacing MNIST to keep the evaluation suite consistently deep (three datasets, three seeds, significance testing) within available compute. A large-scale real-world federated benchmark (e.g., FedScale, LEAF) was not feasible within the revision window and is noted as a direction for future work.

Location of change: "Dataset Profiles" subsection; Conclusion (future work).

Comment: 6. Reported improvements should be supported with statistical significance analysis and confidence intervals.

Response:

We report paired t-tests (new Table 2) across n=3 independent seeds for every FedHST-vs-baseline comparison, together with mean ± standard deviation (from which confidence intervals can be directly derived) rather than explicit CI bounds, to keep the tables compact. We are happy to add explicit 95% CIs if the reviewer considers this necessary.

Location of change: New Table 2 ("Paired significance tests"); Table 1 (mean ± std).

Response to Reviewer #3

Comment: 1. Relatively small benchmark datasets; validation on real-world federated datasets is necessary.

Response:

As above (Reviewer #2, point 5): CIFAR-100 added in place of MNIST; a real-world federated dataset was not feasible within the revision window and is noted as a limitation and future-work direction.

Location of change: "Dataset Profiles" subsection; Conclusion.

Comment: 2. Recent SOTA methods are missing.

Response:

FedYogi, FedAdagrad, FedProx, and SCAFFOLD are now contextualized in Related Work (please see response to Editor Comment 1), while FedDyn is evaluated empirically across all three datasets as the strongest and most theoretically distinct baseline.

Location of change: "Algorithmic Baselines" subsection; new Table 1.

Comment: 3. Ablation study is missing; ablation with different spatial damping parameters is required.

Response:

Added, including the specific γ sweep requested (new Table 4), plus four additional ablation dimensions (Tables 5-8).

Location of change: "Ablation Study" subsection (new Tables 4-8).

Comment: 4. Server-side computational overhead calculation is missing.

Response:

Added: new Table 3 reports measured wall-clock server-side (and client-side) time per round for FedHST, FedAdam, and FedDyn, and the theoretical communication payload.

Location of change: "Computational and Communication Overhead" subsection (new Table 3).

Summary of New/Changed Manuscript Elements

All new or substantively revised content is highlighted in blue in the accompanying "Revised Manuscript with Track Changes" file. In summary, the revision: replaces MNIST with CIFAR-100 and concentrates the empirical comparison on FedAdam and FedDyn (with FedAvgM, FedYogi, FedAdagrad, FedProx, and SCAFFOLD discussed in Related Work and tested on FashionMNIST during revision, per the rationale in our response to Editor Comment 1); reports results over three independent seeds with paired statistical significance testing across FashionMNIST, CIFAR-10, and CIFAR-100; adds a five-dimension ablation study (γ, β1/β2, local epochs, non-IID severity, client count/participation ratio); adds a computational and communication overhead analysis; adopts a lighter, Group-Normalized residual network architecture with reduced communication payload for CIFAR-10/CIFAR-100; shortens and tone-moderates the Introduction and Related Work; and adds a Data Availability Statement and Funding Statement.

We thank the Editor and Reviewers again for their time and constructive guidance, which we believe has meaningfully improved the manuscript.

Sincerely,

Duy-Dong Le and Thai-Thinh Dang

University of Economics Ho Chi Minh City (UEH), Vietnam

Attachment Submitted filename: Response_to_Reviewers.docx

# Editor report: Decision Letter 1

**DOI:** 10.1371/journal.pone.0360546.r003

**Authors:** Debotosh Bhattacharjee

28 Sep 2026

FedHST: Federated Hybrid Spatial–Temporal Optimization for Robust Non-IID Learning

PONE-D-26-28169R1

Dear Dr. Le,

We’re pleased to inform you that your manuscript has been judged scientifically suitable for publication and will be formally accepted for publication once it meets all outstanding technical requirements.

Within one week, you’ll receive an e-mail detailing the required amendments. When these have been addressed, you’ll receive a formal acceptance letter and your manuscript will be scheduled for publication.

An invoice will be generated when your article is formally accepted. Please note, if your institution has a publishing partnership with PLOS and your article meets the relevant criteria, all or part of your publication costs will be covered. Please make sure your user information is up-to-date by logging into Editorial Manager at Editorial Manager® and clicking the ‘Update My Information' link at the top of the page. For questions related to billing, please contact billing support.

If your institution or institutions have a press office, please notify them about your upcoming paper to help maximize its impact. If they’ll be preparing press materials, please inform our press team as soon as possible -- no later than 48 hours after receiving the formal acceptance. Your manuscript will remain under strict press embargo until 2 pm Eastern Time on the date of publication. For more information, please contact onepress@plos.org.

Kind regards,

Debotosh Bhattacharjee, PhD

Academic Editor

PLOS One

Additional Editor Comments (optional):

The authors have taken care of review comments successfully.

Reviewers' comments:

# Editor report: Acceptance letter

**DOI:** 10.1371/journal.pone.0360546.r004

**Authors:** Debotosh Bhattacharjee

PONE-D-26-28169R1

PLOS One

Dear Dr. Le,

I'm pleased to inform you that your manuscript has been deemed suitable for publication in PLOS One. Congratulations! Your manuscript is now being handed over to our production team.

At this stage, our production department will prepare your paper for publication. This includes ensuring the following:

* All references, tables, and figures are properly cited

* All relevant supporting information is included in the manuscript submission,

* There are no issues that prevent the paper from being properly typeset

You will receive further instructions from the production team, including instructions on how to review your proof when it is ready. Please keep in mind that we are working through a large volume of accepted articles, so please give us a few days to review your paper and let you know the next and final steps.

Lastly, if your institution or institutions have a press office, please let them know about your upcoming paper now to help maximize its impact. If they'll be preparing press materials, please inform our press team within the next 48 hours. Your manuscript will remain under strict press embargo until 2 pm Eastern Time on the date of publication. For more information, please contact onepress@plos.org.

You will receive an invoice from PLOS for your publication fee after your manuscript has reached the completed accept phase. If you receive an email requesting payment before acceptance or for any other service, this may be a phishing scheme. Learn how to identify phishing emails and protect your accounts at https://explore.plos.org/phishing.

If we can help with anything else, please email us at customercare@plos.org.

Thank you for submitting your work to PLOS One and supporting open access.

Kind regards,

PLOS One Editorial Office Staff

on behalf of

Dr. Debotosh Bhattacharjee

Academic Editor

PLOS One
